# SS-31 alleviated nociceptive responses and restored mitochondrial function in a headache mouse model via Sirt3/Pgc-1α positive feedback loop

**DOI:** 10.1186/s10194-023-01600-6

**Published:** 2023-06-05

**Authors:** Zhengming Shan, Yajuan Wang, Tao Qiu, Yanjie Zhou, Yu Zhang, Luyu Hu, Lili Zhang, Jingjing Liang, Man Ding, Shanghua Fan, Zheman Xiao

**Affiliations:** 1 Department of Neurology, Renmin Hospital of Wuhan University, 99 Zhang Zhidong Road, Wuchang District, Wuhan, 430060 Hubei Province China; 2 Central Laboratory, Renmin Hospital of Wuhan University, 9 Zhang Zhidong Road, Wuchang District, Wuhan, 430060 Hubei Province China

**Keywords:** SS-31, Migraine, Mitochondrial dysfunction, Mitochondrial homeostasis, Sirt3, Pgc-1α

## Abstract

Migraine is the second highest cause of disability worldwide, bringing a huge socioeconomic burden. Improving mitochondrial function has promise as an effective treatment strategy for migraine. Szeto-Schiller peptide (SS-31) is a new mitochondria-targeted tetrapeptide molecule that has been shown to suppress the progression of diseases by restoring mitochondrial function, including renal disease, cardiac disease, and neurodegenerative disease. However, whether SS-31 has a therapeutic effect on migraine remains unclear. The aim of this study is to clarify the treatment of SS-31 for headache and its potential mechanisms. Here we used a mouse model induced by repeated dural infusion of inflammatory soup (IS), and examined roles of Sirt3/Pgc-1α positive feedback loop in headache pathogenesis and mitochondrial function. Our results showed that repeated IS infusion impaired mitochondrial function, mitochondrial ultrastructure and mitochondrial homeostasis in the trigeminal nucleus caudalis (TNC). These IS-induced damages in TNC were reversed by SS-31. In addition, IS-induced nociceptive responses were simultaneously alleviated. The effects of SS-31 on mitochondrial function and mitochondrial homeostasis (mainly mitochondrial biogenesis) were attenuated partially by the inhibitor of Sirt3/Pgc-1α. Overexpression of Sirt3/Pgc-1α increased the protein level of each other. These results indicated that SS-31 alleviated nociceptive responses and restored mitochondrial function in an IS-induced headache mouse model via Sirt3/Pgc-1α positive feedback loop. SS-31 has the potential to be an effective drug candidate for headache treatment.

**Graphical Abstract:**

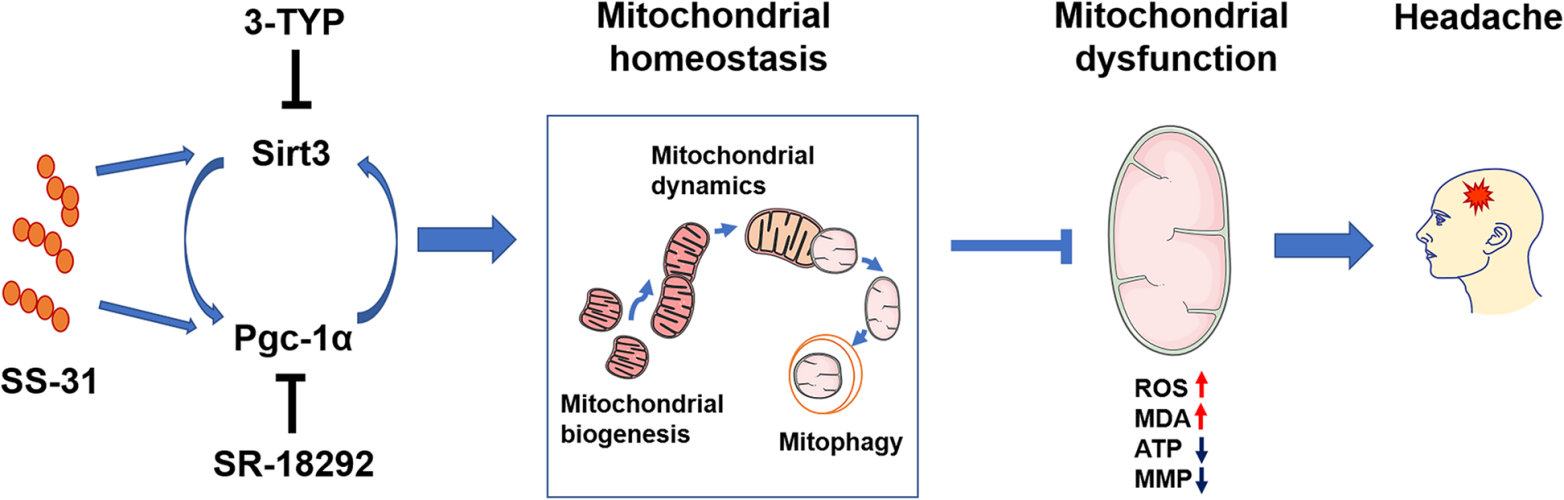

**Supplementary Information:**

The online version contains supplementary material available at 10.1186/s10194-023-01600-6.

## Introduction

Migraine is a common neurological disorder manifested by recurrent attacks of headache and accompanying symptoms, such as nausea and vomiting [1]. As the second highest cause of disability worldwide [2], migraine leads to a huge socioeconomic burden. A recent review proposed that migraine is a response to high cerebral oxidative stress levels and insufficient energy production with mitochondrial dysfunction [3, 4]. ^31^P-nuclear magnetic resonance studies revealed a lower phosphocreatine to creatine ratio and increased levels of adenosine diphosphate (ADP) in patients with migraine [5]. Moreover, adenosine triphosphate (ATP) levels decreased in the occipital cortex of patients with migraine [6]. Of note, several nutrients targeting energy production and mitochondrial metabolism have shown efficacy in preventing migraine, such as riboflavin, coenzyme Q_10_, thiamine and niacin [7–9]. In migraine rodent models, mitochondrial dysfunction was also observed [10]. Thus, improving mitochondrial function has promise as an effective treatment for migraine.

Mitochondrial homeostasis, a process to maintain mitochondrial function, is achieved through appropriate coordination among mitochondrial biogenesis, mitochondrial dynamics and mitophagy [11]. Under certain conditions, for instance, exercise, mitochondrial biogenesis is initiated to make new mitochondria by increasing the expression of peroxisome proliferator-activated receptor-gamma co-activator 1α (Pgc-1α) [12]. The damaged mitochondria are diluted and cleared through mitochondrial dynamics and mitophagy [11]. Impaired mechanisms of maintaining mitochondrial homeostasis can cause an imbalance of mitochondrial metabolism and a range of pathological conditions. A recent study showed that mitochondrial biogenesis was injured and mitochondrial dynamics was altered in migraine rat models [13], suggesting the imbalance of mitochondrial homeostasis may be a potential mechanism driving mitochondrial dysfunction in migraine.

Sirtuin 3 (Sirt3), one of the most prominent protein deacetylases, is mainly localized in mitochondria and participates in the maintenance of mitochondrial homeostasis, through increasing mitochondrial biogenesis [14], remodeling mitochondrial dynamics [15] and regulating mitophagy [16]. Pgc-1α may be another potential key target for mitochondrial homeostasis. The mainstream view is that Pgc-1α functions as a key regulator of mitochondrial biogenesis [12], while some studies have demonstrated that Pgc-1α also controls mitochondrial dynamics [17, 18] and regulates mitophagy [19].

Mounting studies have shown that Pgc-1α has dense connections to Sirt3. Pgc-1α, functioning as a transcriptional coactivator, can increase the expression of Sirt3 [20, 21]. Moreover, Sirt3 has been shown to promote the expression of Pgc-1α [22]. However, the associated-mechanisms remain controversial. It seems that the two proteins form a positive feedback loop [23], which maintains mitochondrial homeostasis and regulates mitochondrial function.

Szeto-Schiller peptide (SS-31, also called elamipretide), a mitochondria-targeted tetrapeptide molecule, is able to cross the cell membrane without relying on the mitochondrial transmembrane potential (MMP) and accumulates in the mitochondrial inner membrane specifically [24]. By selectively binding to cardiolipin which is uniquely expressed on the mitochondrial inner membrane, the SS-31 modulates the hydrophobic interaction between cytochrome c and cardiolipin and promote the electron carrier properties [25]. In several human diseases, SS-31 is confirmed to have therapeutic effects on the imbalance of mitochondrial metabolism, mitochondrial homeostasis and disease progression, including renal disease [26], cardiac disease [27] and neurodegenerative disease [28]. Until now, no study has investigated whether SS-31 has a therapeutic effect on migraine.

Thus, we proposed the following hypotheses according to above evidence: the mitochondria-targeted antioxidant peptide SS-31 alleviated nociceptive responses and restored mitochondrial function in a headache mouse model via mitochondrial homeostasis regulated by Sirt3/Pgc-1α positive feedback loop. In this study, we used an IS-induced headache mouse model to investigate the effect of SS-31 on headache. Moreover, inhibitors of Sirt3 and Pgc-1α were administered to research possible mechanisms. By overexpression of Sirt3/Pgc-1α in PC12 cells, we further explored whether a Sirt3/Pgc-1α positive feedback loop existed.

## Materials and methods

### Animals

A total of 221 male C57BL/6 mice and 12 female mice weighing 20–25 g were used for this study. The sample size was calculated according to the variance of pilot studies and previous experiments. The animals were housed at 22 ± 2 ℃ under 12 h light/dark cycle, and had free access to food and water. All animals were adapted to experimental environment for 7 days before the surgical procedure. The experiments were approved by the Institutional Animal Care and Use Committee (IACUC) of Renmin Hospital of Wuhan University (IACUC Issue No. WDRM20201205). All experimental procedures were conducted following the recommendations of the International Association for the Study of Pain in Conscious Animals. To minimize the number of animals, the left and right trigeminal nucleus caudalis (TNC) of the same mouse were respectively used to perform different experiments.

### Headache model establishment

The headache animal model was established by repeated infusion of IS to the dura. The surgical procedures were performed following a previous report [29]. In brief, under isoflurane anesthesia (3% isoflurane for induction and 1.5% isoflurane for maintenance), a midline incision was performed to expose the skull clearly. Then, a skull drill (Reward 87001) was used to make a 1 mm diameter cranial window in the right frontal bone (+ 1.0 mm after the bregma and + 1.0 mm lateral to the bregma). We were careful to avoid damaging the dura. Afterwards, a guide cannula was implanted above the dura and affixed to the skull, to deliver inflammatory soup (IS) or saline to the dura of animals. To minimize the extent of suffering, we reduced the length of the skin incision as much as possible and gave post-surgical anti-infection and analgesia treatment. The incision was sutured and recovered for 3 days, then subsequent experiments were carried out.

### Drug administration

According to a previous study [29], IS (including 2 mM serotonin, 2 mM histamine, 2 mM bradykinin, and 0.2 mM prostaglandin E2 in saline) was administered to the dura of animals by a microinjector. SS-31 (Qiangyaobio, Shanghai, China), a mitochondrial targeting peptide, was dissolved in phosphate buffered saline (PBS) to a concentration of 5 mg/kg [30]. 3-TYP (50 mg/kg, MCE, America) [31], a selective Sirt3 inhibitor, was dissolved in a 5% DMSO solution. SR-18292 (45 mg/kg, MCE, America) [4], a Pgc-1α inhibitor, was dissolved in a 5% DMSO solution. SS-31, 3-TYP and SR-18292 were all administered intraperitoneally, and their dosages were determined based on previous studies.

### Experiment design

#### Assessment of the mitochondrial function and mitochondrial homeostasis in the IS-induced mouse model

To assess the effect of IS on mitochondrial function, mitochondria ultrastructure and mitochondrial homeostasis, a total of 44 male mice and 12 female mice were randomly divided into 2 groups, respectively: (1) sham and (2) IS. 20 μL IS was delivered to the dura of mice in IS group at the same time each day for 7 consecutive days. The volume of IS was determined according to our previous study [32]. Mice in the sham group received 20 μL saline once daily. After sacrificed, the TNC tissues of mice were collected for next experiments.

#### Evaluation of the effects of SS-31 for nociceptive responses, mitochondrial function and mitochondrial homeostasis

A total of 72 male mice were randomly divided into 3 groups: (1) sham, (2) IS and (3) SS-31. Daily dural injection of 20μL IS for the IS and SS-31 group mice was performed. Mice in the SS-31 group received intraperitoneal injection (i.p.) of SS-31 30 min before IS injection daily for 7 consecutive days. Mice in the sham and IS group received intraperitoneal injection of equivalent volume of PBS. Detailed drug regimen was shown in Fig. 2A.

#### Investigation into whether the effects of SS-31 were mediated by Sirt3/Pgc-1α positive feedback

All 105 male mice were randomly divided into 5 groups: (1) PBS + sham + DMSO, (2) PBS + IS + DMSO, (3) SS-31 + IS + DMSO, (4) SS-31 + IS + 3-TYP and (5) SS-31 + IS + SR-18292. Mice in PBS + IS + DMSO, SS-31 + IS + DMSO, SS-31 + IS + 3-TYP and SS-31 + IS + SR-18292 groups received IS dural injections. SS-31 was intraperitoneally administered 30 min before IS infusion in mice of SS-31 + IS + DMSO, SS-31 + IS + 3-TYP and SS-31 + IS + SR-18292 groups. Mice in SS-31 + IS + 3-TYP group received 3-TYP (i.p.) 30 min after IS infusion. Mice in SS-31 + IS + SR-18292 group received SR-18292 (i.p.) 30 min after IS infusion. All drugs were administered at the same time each day for 7 consecutive days. Detailed drug regimen was shown in Fig. 5A.

### Animal behavioral test

The nociceptive behavior of animals was evaluated by head scratching, the periorbital mechanical threshold and the thermal withdrawal latency of the hind paw. After the IS (or saline) injection, we immediately recorded the number of head scratching for 1 h, which represented a nociceptive behavior resulting by IS. The periorbital mechanical threshold was evaluated by a von Frey test, using an up-and-down approach according to previous report [33, 34]. A positive reaction was defined as rapid withdrawal of the head. The nociceptive threshold was recorded as the least force when 3 positive reactions appeared within 5 stimuli. The thermal withdrawal latency of the hind paw was tested by a special heating device, which can control the temperature at around 55 °C. Before being tested, animals were placed at the heating device some times to adapt the environment. Subsequently, we recorded the time from the hind paw touching the heating device to the quick withdrawal of the hind paw from the heating device. We repeated the test three times, with a 3-min interval between each. The average value of the three tests was recorded as the paw withdrawal latency.

### Cell cultures and drug treatment

Rat adrenal medullary pheochromocytoma PC12 cells (purchased from China Center for Type Culture Collection, Wuhan, China) were cultured in complete RPMI-1640 medium containing 10% horse serum, 5% fetal bovine serum and 1% penicillin/streptomycin. All cells were incubated at 37 °C in a 5% CO_2_ incubator. Differentiation medium, which consisted of RPMI-1640 medium, 1% horse serum and 100 ng/mL nerve growth factor (NGF, Thermo Fisher, America), was used to induce differentiation. The cells between 10^th^ and 20^th^ generations were seeded in plates or dishes coated with polylysine (0.1 mg/mL) and cultured in differentiation medium for 10 days. After 2-h treatment of SS-31 (100 nM, dissolved in PBS), H_2_O_2_ (300 μM) was added into the medium. The cells were collected 12 h later for western blot analysis and flow cytometric analysis. Details of the 293 T cell culture conditions are provided in the supplementary materials.

### Lentivirus construction and transfection

The lentivirus (LV) overexpressing Sirt3 (GV358, Ubi-MCS-3FLAG-CBh-gcGFP-IRES-puromycin), Pgc-1α (GV492, Ubi-MCS-3FLAG-CBh-gcGFP-IRES-puromycin) and the control LV (CON335) were designed and synthesized by Shanghai Gene Pharma Co. Ltd. Infected cells with puromycin resistance were selected using puromycin (Beyotime, Shanghai, China). Then the infected cells were seeded in 6-well plates. After 12 h of treatment with/without H_2_O_2_ (300 μM), cells were collected for western blot analysis, flow cytometric analysis and ATP level detection.

### Western blot analysis

Tissues or cells for western blot analysis were homogenized in ice-cold lysis buffer, which contains RIPA, cocktail and phenylmethylsulfonyl fluoride (PMSF). Then, homogenates were centrifuged to isolate total protein. Specially, a cell mitochondria isolation kit (Beyotime) was used to isolate mitochondria for clarifying the localization of Sirt3 and Pgc-1α. Protein concentration was measured using a bicinchonic acid (BCA) assay kit (Beyotime). After mixed with loading buffer, the lysates were heated for 5–10 min at 100 °C. Subsequently, equal amounts of protein from different samples were separated by 10% SDS polyacrylamide gel (Bio-Rad) and transferred to polyvinylidene-difluoride (PVDF) membranes (Millipore, Billerica, America). After being blocked with 5% skim milk for 1 h and washed with TBST, the membranes were incubated with the primary antibody overnight at 4 °C. The target protein information of the specific primary antibody was used as follows: Pgc-1α (1:1000, Novus Biologicals), mitochondrial transcription factor A (Tfam, 1:1000, Abcam), mitofusin 2 (Mfn2, 1:5000, Proteintech), fission 1 (Fis1, 1:2000, Wuhan Fine bio), dynamin related protein 1 (Drp1, 1:2000, Wuhan Fine bio), Beclin1 (1:1000, Wanleibio), PTEN-induced putative kinase 1 (Pink1, 1:1000, Wanleibio), Parkin (1:1000, Wanleibio), P62 (1:1000, Cell Signaling Technology), c-fos (1:5000, Proteintech), Sirt3 (1:1000, Novus Biologicals/ Wanleibio), proliferating cell nuclear antigen (Pcna, 1:500, Wanleibio), cytochrome oxidase subunit IV (CoxIV, 1:5000, Proteintech) and β-actin (1:3000, Servicebio). After washing with TBST, the membranes were incubated with the corresponding horseradish peroxidase (HRP) labeled secondary antibody (1:5000, Servicebio) for 1 h at room temperature (RT). Then, the membranes were washed with TBST and visualized using Odyssey CLx Image Studio 3. The gray intensity of strips was analyzed with Image J 1.52 (National Institutes of Health). The protein level was normalized using β-actin as the internal reference.

### Immunofluorescence staining

Animals were transcardially perfused with 0.9% saline under deep anesthesia, and then with 4% paraformaldehyde in 0.1 M phosphate buffer (pH 7.4). The brain was separated immediately and postfixed in 4% paraformaldehyde for 24 h. Soon afterwards, the brain tissue was cut into sections (5 μm) after paraffin-embedded. Paraffin-embedded brain sections or cell climbing slices were washed with 0.1 M PBS and incubated with 0.3% triton X-100 (Servicebio) for 10 min. Then after washed with PBS again, the sections were blocked with 5% bovine serum albumin (BSA) and incubated with the primary antibody overnight at 4 °C, including c-fos (1:200, Servicebio), NeuN (1:200, Proteintech), voltage-dependent anion channel 1 (VDAC1, 1:200, Abcam), Calcitonin gene-related peptide (CGRP, 1:200, Santa-Cruz), Sirt3 (1:200, Novus Biologicals) and Pgc-1α (1:200, Novus Biologicals). The next day, the sections were incubated with the Cy3-/FITC-labeled anti-mouse/rabbit antibody (1:200, Servicebio) for 1 h at RT. Then the nuclei were stained by DAPI. Afterwards, images in the TNC (as shown in Supplementary Fig. 1E) were captured with an ECLIPSE Ti-U microscope/ FV1200 confocal microscopy (Olympus, Japan) using the NLS-Elements BR.3.0 software (Nikon, Melville, NY).

### Coimmunoprecipitation (co-IP)

Co-IP was performed in PC12 cells infected by LV overexpressing Sirt3-Flag to explore protein–protein interaction using anti-Flag magnetic beads (Beyotime) on the basis of manufacturer’s instructions. Briefly, cells were lysed with lysis buffer on ice. After centrifugation, the supernatant was collected and incubated with magnetic beads (IgG magnetic beads were used as negative control) for 2 h at room temperature. Then, magnetic beads were eluted by SDS-PAGE sample buffer. The proteins in elution buffer were analyzed by western blot.

### Transmission electron microscope (TEM)

Animals were deeply anesthesia and perfused with 0.9% saline followed by glutaraldehyde for fixation. Then the brain was separated immediately. The TNC tissues (as shown in Supplementary Fig. 1E) were cut into 2 mm^2^ pieces in cold glutaraldehyde and incubated in 4% glutaraldehyde at 4 °C for 24 h. Embedding, sectioning, staining and image capturing were performed in The Electric Mirror Center of the Renmin Hospital of Wuhan University.

### Measurement of reactive oxygen species (ROS) levels

The ROS levels of animal tissues was determined with a ROS enzyme linked immunosorbent assay (ELISA) kit (Saipeibio, Wuhan, China), according to the protocol. Briefly, brain tissues were homogenized in PBS. After centrifuging at 13,000 rpm for 15 min at 4 °C, the supernatants were removed from the lysates for ROS detection using the ELISA kit. Then, a multifunctional microplate reader (EnSight, Perkin Elmer, America) was used to measure absorbance at 450 nm. Protein concentration measured by BCA assay kit (Beyotime) was used to normalize ROS results.

Using MitoSOX Red reagent (Yeasen Bio, Shanghai, China), mitochondrial ROS levels of PC12 cells were measured. According to the instruction manuals, after incubated with 2 μM MitoSox Red reagent for 30 min, the ROS levels were detected using a flow cytometer (Beckman Coulter, America).

### Measurement of malondialdehyde (MDA) and ATP levels

Lipid peroxidation MDA levels were detected with a lipid peroxidation MDA assay kit (Beyotime). In short, samples were processed according to the instruction manual, and the MDA concentration was measured by a multifunctional microplate reader at 450 nm. The final MDA levels were normalized by the protein concentrations.

The ATP concentration was determined by using an ATP assay kit (Beyotime) by a multifunctional microplate reader (using luminometer) according to the manufacturer’s protocol. Briefly, fresh tissues after sacrificed or PC12 cells were collected and homogenized in lysates on ice, in which condition ATP remains stable for at least 6 h according to the manufacturer’s protocol. After centrifuging at 12,000 g for 5 min at 4 °C, the supernatants were removed for ATP detection. The final ATP levels were normalized by protein concentrations.

### Detection of MMP levels

The fresh tissues were separated quickly. Then functional mitochondria were extracted with a tissue mitochondrial isolation kit (Beyotime). Next, the MMP level was determined using a tetramethylrhodamine ethyl ester perchlorate (TMRE) by a mitochondrial membrane potential assay kit (Beyotime). Meanwhile, the protein concentration was detected using a Bradford assay kit (Servicebio). The MMP level was normalized by the protein concentration.

For PC12 cells, after incubated with 5 μM TMRE for 15 min, cells were collected, and the MMP levels were detected using a flow cytometer (Beckman Coulter, America).

### Statistical analysis

GraphPad Prism 8 (San Diego, CA) was used to generate the graphs, and statistical analysis was performed by SPSS 22.0 (Chicago, IL, USA). The results are expressed as the means ± SD. The parametric Student’s *t*-test was used to analyze the differences between two independent groups. One-way ANOVA with Tukey’s post-hoc test was used for more groups. Two-way ANOVA with the Bonferroni post-hoc test was used for behavioral data analysis. *p* < 0.05 was considered statistically significant.

## Results

### Mitochondria were injured and mitochondrial homeostasis was unbalanced after repeated IS infusion in a headache mouse model

#### Nociceptive responses were activated by repeated IS infusion

We established a headache mouse model by IS dural infusion for 7 consecutive days, and behavioral tests were conducted every day after IS infusion. In male mice, as shown in Supplementary Fig. 1A, the number of head scratching in 1-h after IS infusion increased significantly on day 5, 6 and 7 in IS group compared to the sham group. The periorbital mechanical threshold decreased markedly on day 3, 4, 5, 6 and 7 in the IS group compared to the sham group (Supplementary Fig. 1B). Western blot analysis (Supplementary Fig. 1C) and immunofluorescence staining (Supplementary Fig. 1D-F) showed that expression of c-fos increased significantly in TNC after repeated IS infusion. Consistently, after repeated IS infusion, the number of head scratching in 1-h and the expression of c-fos increased, the periorbital mechanical threshold and the paw withdrawal latency decreased markedly in the female mouse model (Supplementary Fig. 5A-B). These results indicated that repeated IS infusion activated nociceptive responses in a headache mouse model.

#### Repeated IS infusion disrupted the normal function of the mitochondria

**Fig. 1:**
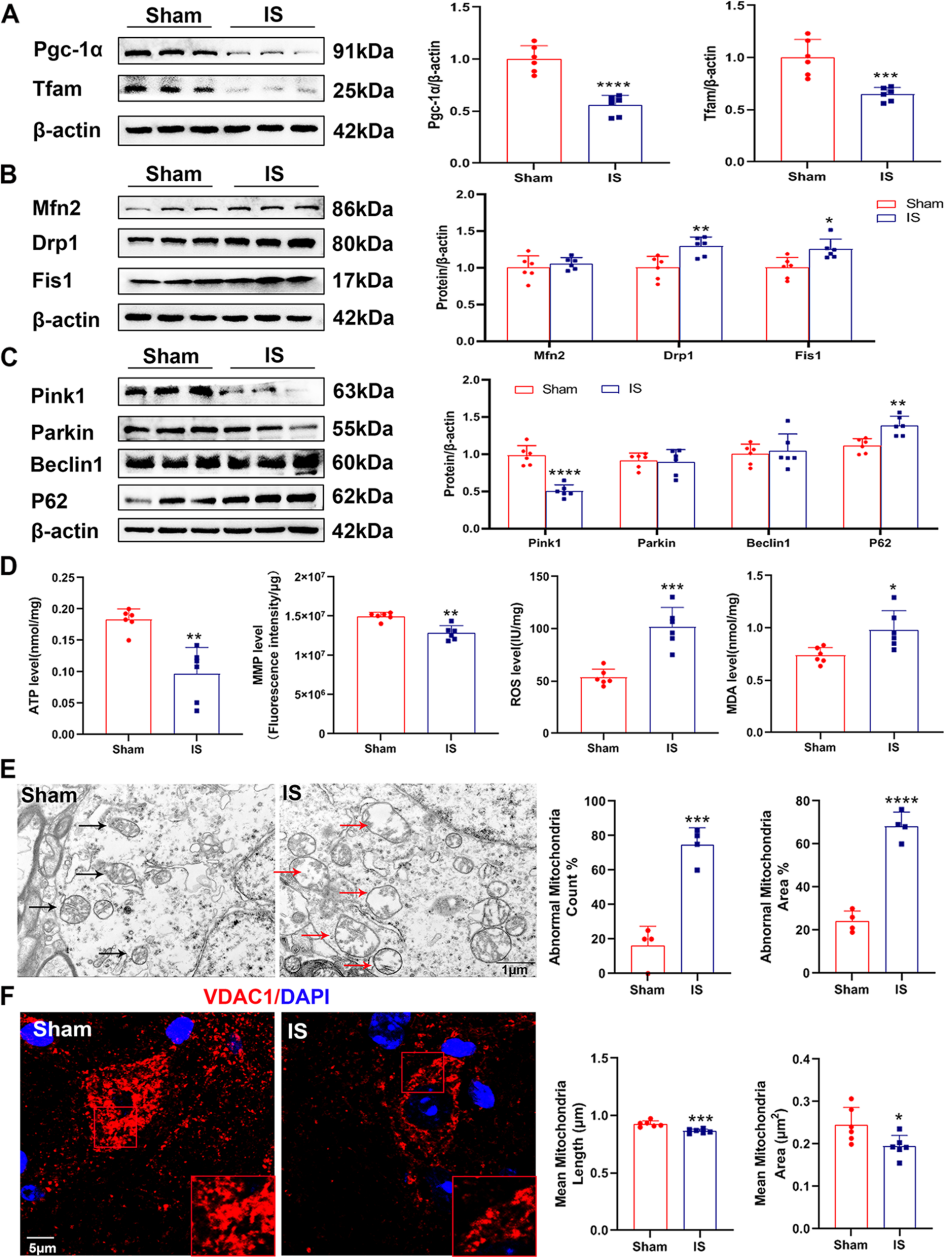
Mitochondria were injured and mitochondrial homeostasis was altered after repeated IS infusion in a headache male mouse model. Male C57BL/6 mice were sham treated or dural-infused of inflammatory soup (IS) for 7 consecutive days, and then sacrificed for mitochondrial assessment. **A**-**C** Representative immunoblots and quantification of the protein levels of Pgc-1α (^****^p < 0.0001), Tfam (^***^p = 0.0009), Drp1 (^**^p = 0.0050), Mfn2, Fis1 (^*^p = 0.0108), Beclin1, P62 (^**^p = 0.0021), Pink1 (^****^p < 0.0001), and Parkin in the TNC. *n* = 6 per group; Student’s *t*-test. **D** The levels of ATP (^**^p = 0.0022), MMP (^**^p = 0.0043), ROS (^***^p = 0.0002) and MDA (^*^p = 0.0136) were detected and normalized by total protein concentrations in the TNC. *n* = 6 per group; Student’s *t*-test. **E** Mitochondrial ultrastructure in the TNC by TEM analysis. The abnormal mitochondria count percent (^***^p = 0.0002) and area percent (^****^p < 0.0001) were calculated. Red arrowhead, damaged mitochondria (swollen mitochondria and reduction of mitochondrial cristae). Black arrowhead, normal mitochondria. Scale bar, 1 μm. *n* = 4 per group; Student’s *t*-test. **F** Immunofluorescence staining of VDAC1 and nucleus (DAPI) in the TNC. The mean mitochondria length (^***^p = 0.0006) and area (^*^p = 0.0285) were calculated. Scale bar, 5 μm. *n* = 6 per group; Student’s *t*-test. Data are represented as Mean ± SD; ^*^p < 0.05, ^**^p < 0.01, ^***^p < 0.001 and ^****^p < 0.0001 as compared to sham group

Then, TNC tissues of mice were collected to analyze mitochondrial function. In male mice, after repeated dural IS stimulation, the MMP levels and ATP levels decreased significantly in the TNC of the IS group compared to the sham group (Fig. 1D). The ROS levels and the MDA levels were markedly higher in the IS group than sham group (Fig. 1D). Consistently, the ATP level decreased significantly after repeated IS infusion in the female mouse model (Supplementary Fig. 5D). Our findings suggested that mitochondrial function was disrupted by repeated IS infusion.

#### Mitochondrial homeostasis was unbalanced after repeated IS infusion

Mitochondrial biogenesis is a process which new mitochondria are produced from pre-existing ones [12]. We detected Pgc-1α and Tfam expressions by western blot analysis (Fig. 1A and Supplementary Fig. 5E) to assess mitochondrial biogenesis. Our results showed that the protein levels of Pgc-1α and Tfam decreased significantly in IS group compared to the sham group in both male and female mouse models.

Mitochondrial fusion is mediated by optic atrophy 1 (Opa1) and Mfn 1/2. Meanwhile, recruitment of Drp1 from the cytosol to the outer mitochondrial membrane by Fis1 drives mitochondrial fission [35]. We examined whether mitochondrial dynamics was altered after repeated IS infusion by detecting the protein levels of Mfn2, Drp1 and Fis1. As shown in Fig. 1B and Supplementary Fig. 5F, in both male and female mouse models, the protein levels of Drp1 and Fis1 increased significantly in the IS group compared to the sham group, while Mfn2 had no significant change in the two groups. VDAC1, located in the mitochondrial outer membrane, was used to represent mitochondrial morphology according to a previous study [36]. As shown in Fig. 1F, the mean mitochondria length and area in the IS group were markedly lower than the sham group. This result indicated that there were more fragmented mitochondria in the TNC of IS group, while the sham group had more filamentous mitochondria. Moreover, TEM analysis (Fig. 1E) showed that the mitochondrial ultrastructure was impaired in the TNC of the IS group with mitochondria swollen and less cristae remained. The abnormal mitochondria count percent and area percent significantly increased after repeated IS infusion. These results indicated that mitochondrial dynamics was altered, and mitochondrial fragmentation was aggravated after repeated IS infusion.

Mitophagy is a process which damaged mitochondria are recognized and delivered to the lysosome for degradation [11]. We measured the protein levels related to mitophagy, including Pink1, Parkin, P62 and Beclin1 (Fig. 1C and Supplementary Fig. 5G). After repeated IS infusion, in both male and female mouse models, the expression of P62 increased significantly in the TNC of IS group compared to the sham group, while the protein level of Pink1 decreased markedly in the IS group. However, the expression levels of Parkin and Beclin1 had no significant change in the two groups. These results suggested that mitophagy was impaired to a certain extent after repeated IS infusion.

Collectively, repeated IS dural infusion resulted in disruption of mitochondrial function, ultrastructure and homeostasis in the TNC of a headache mouse model.

### SS-31 attenuated IS-induced nociception responses

**Fig. 2:**
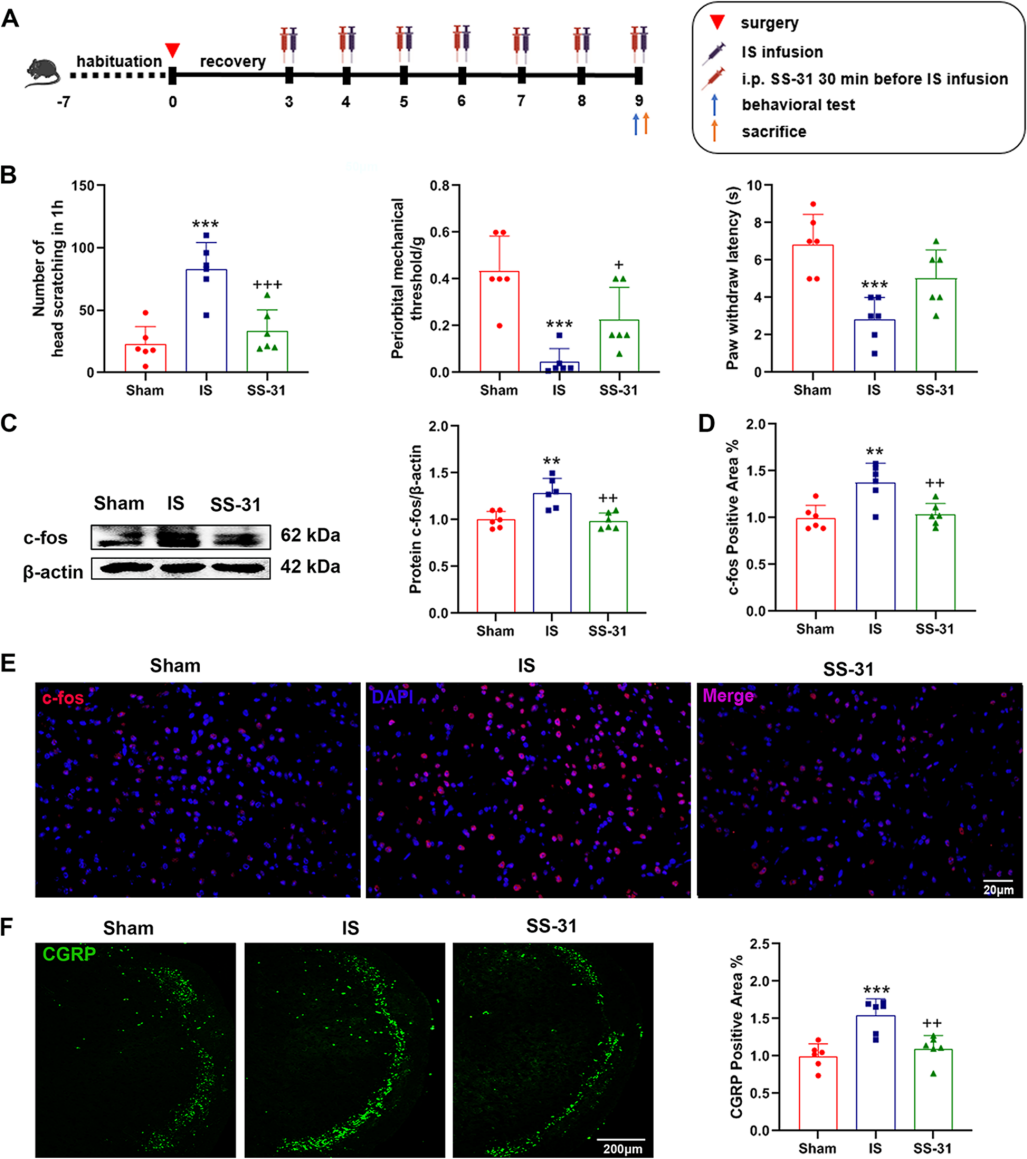
SS-31 attenuated IS-induced nociception responses. **A** Schematic diagram of the experiment. C57BL/6 mice received sham, IS or SS-31 treatment for 7 consecutive days, followed by behavioral tests and sacrifice to evaluate the effects of SS-31 on nociceptive responses. **B** The number of head scratching in 1 h (F(2, 15) = 19.02, ^****^p < 0.0001; ^***^p = 0.001, ^+++^*p* = 0.007), periorbital mechanical threshold (F(2, 15) = 15.14, ^***^p = 0.0003; ^***^p = 0.0002, ^+^*p* = 0.0267) and paw withdrawal latency (F(2, 15) = 11.39, ^***^p = 0.0010; ^***^p = 0.0007) in different groups were recorded. *n* = 6 per group; One-way ANOVA. **C** Western blot analysis of c-fos. Protein level of c-fos was quantified normalized to β-actin in TNC. *n* = 6 per group; One-way ANOVA. F(2, 15) = 12.94, ^***^p = 0.0005; ^**^p = 0.0018, ^++^*p* = 0.0011. **D**-**E** Immunofluorescence staining was used to examine the levels of c-fos in TNC of different groups. Scale bar, 20 μm. *n* = 6 per group; One-way ANOVA. F(2, 15) = 10.41, ^**^p = 0.0015; ^**^p = 0.0025, ^++^*p* = 0.0050. **F** Immunofluorescence staining was used to examine the levels of CGRP in TNC of different groups. Scale bar, 200 μm. *n* = 6 per group; One-way ANOVA. F(2, 15) = 13.96, ^***^p = 0.0004; ^***^p = 0.0005, ^++^*p* = 0.0027. Data are represented as Mean ± SD; ^*^p < 0.05, ^**^p < 0.01 and ^***^p < 0.001 as compared to the Sham group. ^+^*p* < 0.05, ^++^*p* < 0.01 and ^+++^*p* < 0.001 as compared to IS group

To evaluate the treatment of SS-31 on headache, we performed behavioral tests and examined the expressions of c-fos and CGRP. The number of head scratching in 1 h decreased significantly in SS-31 group mice compared to the IS group (Fig. 2B). The periorbital mechanical threshold and paw withdrawal latency were much higher than the IS group (Fig. 2B). Western blot and immunofluorescence analysis showed that the expression levels of c-fos and CGRP decreased in SS-31 group compared to the IS group (Fig. 2C-F). These results demonstrated IS-induced migraine-like behavior (the number of head scratching), hyperalgesia (the periorbital mechanical threshold and the thermal withdrawal latency of the hind paw), the release of CGRP and activation of nociceptive neurons (c-fos) can be attenuated by SS-31.

### SS-31 restored mitochondrial function and mitochondrial homeostasis in an IS-induced headache mouse model and H_2_O_2_-induced PC12 cells

#### Mitochondrial function and mitochondrial homeostasis were restored by SS-31 in an IS-induced mouse model

**Fig. 3:**
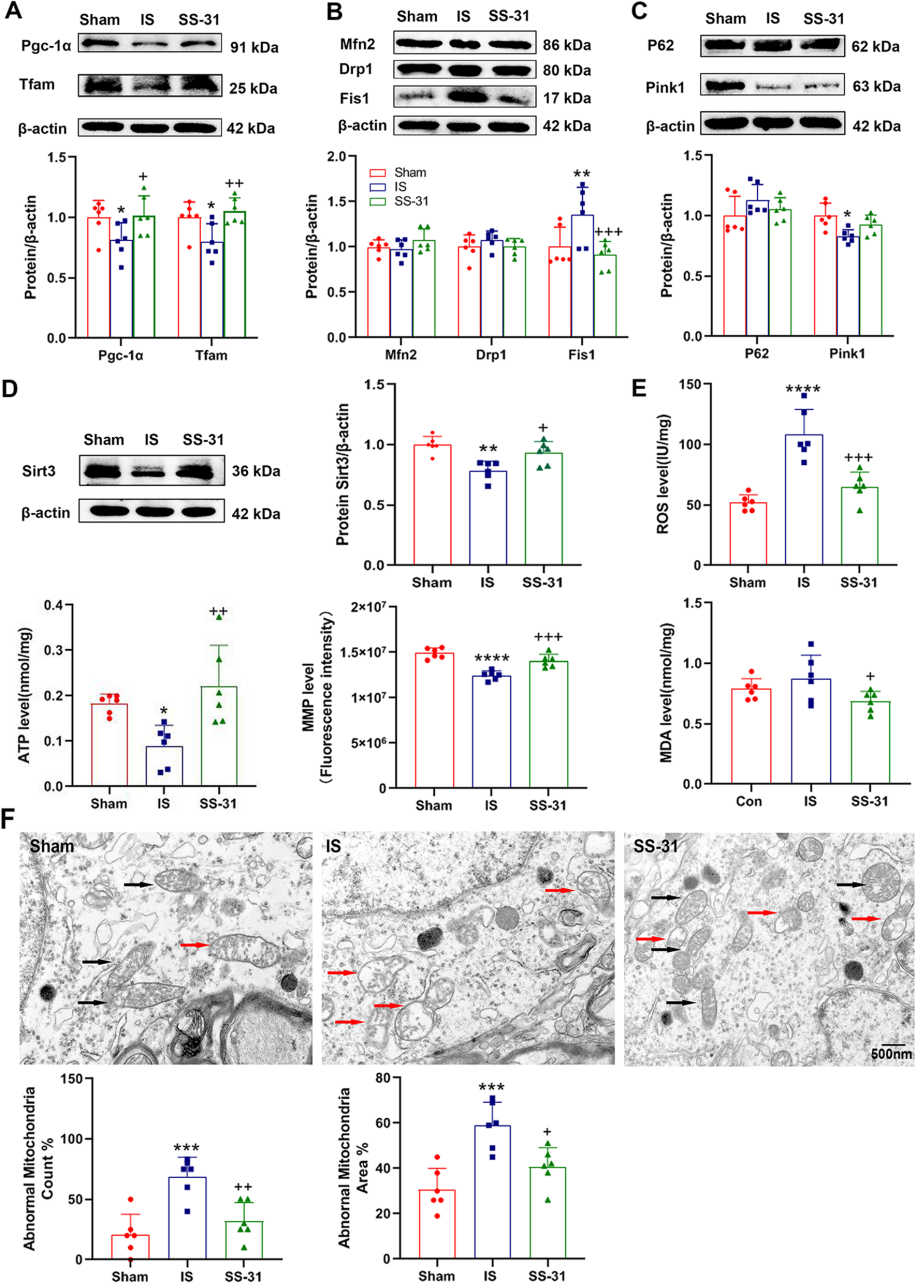
SS-31 restored mitochondrial function and mitochondrial homeostasis in an IS-induced headache mouse model. C57BL/6 mice received sham, IS or SS-31 treatment for 7 consecutive days, and were sacrificed to evaluate the effects of SS-31 on mitochondrial function and mitochondrial homeostasis in TNC. **A**-**C** Western blot analysis was used to asses expression levels of Pgc-1α (F(2, 15) = 5.434, ^*^p = 0.0168; ^*^p = 0.0384, ^+^*p* = 0.0249), Tfam (F(2, 15) = 6.457, ^**^p = 0.0095; ^*^p = 0.0443, ^++^*p* = 0.0100), Mfn2, Drp1, Fis1 (F(2, 15) = 15.74, ^***^p = 0.0002; ^**^p = 0.0015, ^+++^*p* = 0.0003), P62 and Pink1 (F(2, 15) = 5.143, ^*^p = 0.0199; ^*^p = 0.0153) in different groups. *n* = 6 per group; One-way ANOVA. **D** Western blot analysis and quantification of Sirt3 (F(2, 15) = 10.66, ^**^p = 0.0013; ^**^p = 0.0011, ^+^*p* = 0.0208) protein level normalized to β-actin. *n* = 6 per group; One-way ANOVA. **E** The levels of ROS (F(2, 15) = 25.33, ^****^p < 0.0001; ^****^p < 0.0001, ^+++^*p* = 0.0003), ATP (F(2, 15) = 7.704, ^**^p = 0.0050; ^*^p = 0.0413, ^++^*p* = 0.0045), MMP (F(2, 15) = 25.89, ^****^p < 0.0001; ^****^p < 0.0001, ^+++^*p* = 0.0009) and MDA (F(2, 15) = 3.854, ^*^p = 0.0446; ^+^*p* = 0.0362) were detected and normalized by total protein concentrations in different groups. *n* = 6 per group; One-way ANOVA. (F) Mitochondrial ultrastructure in TNC by TEM analysis. The abnormal mitochondria count percent (F(2, 15) = 14.39, ^***^p = 0.0003; ^***^p = 0.0004, ^++^*p* = 0.0034) and area percent (F(2, 15) = 13.70, ^***^p = 0.0004; ^***^p = 0.0003, ^+^*p* = 0.0120) were calculated. Red arrowhead, damaged mitochondria (swollen mitochondria and reduction of mitochondrial cristae). Black arrowhead, normal mitochondria. Scale bar, 500 nm. *n* = 6 per group; One-way ANOVA. Data are represented as Mean ± SD; ^*^p < 0.05, ^**^p < 0.01, ^***^p < 0.001 and ^****^p < 0.0001 as compared to sham group. ^+^*p* < 0.05, ^++^*p* < 0.01 and ^+++^*p* < 0.001 as compared to IS group

To investigate the underlying mechanisms of SS-31 on headache treatment, we assessed mitochondrial function, homeostasis and ultrastructure. The expression of Pgc-1α and Tfam decreased significantly after repeated IS infusion in IS group compared to the sham group, but this change was reversed by SS-31 pretreatment (Fig. 3A). The level of Fis1 protein expression in SS-31 group was much lower than IS group mice, but no significant changes were shown in Mfn2 and Drp1 expression (Fig. 3B). Western blot analysis of P62 and Pink1 showed no significant difference between IS and SS-31 group mice (Fig. 3C). Overall, these results indicated IS-induced imbalance in mitochondrial homeostasis could be restored by SS-31 partially.

The ROS, ATP, MMP and MDA levels were measured to evaluate mitochondrial function. A decrease in ATP content and MMP level induced by repeated IS infusion was reversed by SS-31, while the increase in ROS and MDA levels were eliminated by SS-31 (Fig. 3E). TEM analysis showed more normal mitochondria in SS-31 group while IS group had more swollen and less cristae remained mitochondria (Fig. 3F). The abnormal mitochondria count percent and area percent increased significantly in SS-31 group compared to the sham group.

Furthermore, western blot analysis showed that repeated IS infusion resulted in Sirt3 expression decreased, and SS-31 pretreatment reversed this change (Fig. 3D), suggesting Sirt3 potentially involved in mechanisms of SS-31.

#### Mitochondrial function and mitochondrial homeostasis were restored by SS-31 in H_2_O_2_-induced PC12 cells

**Fig. 4:**
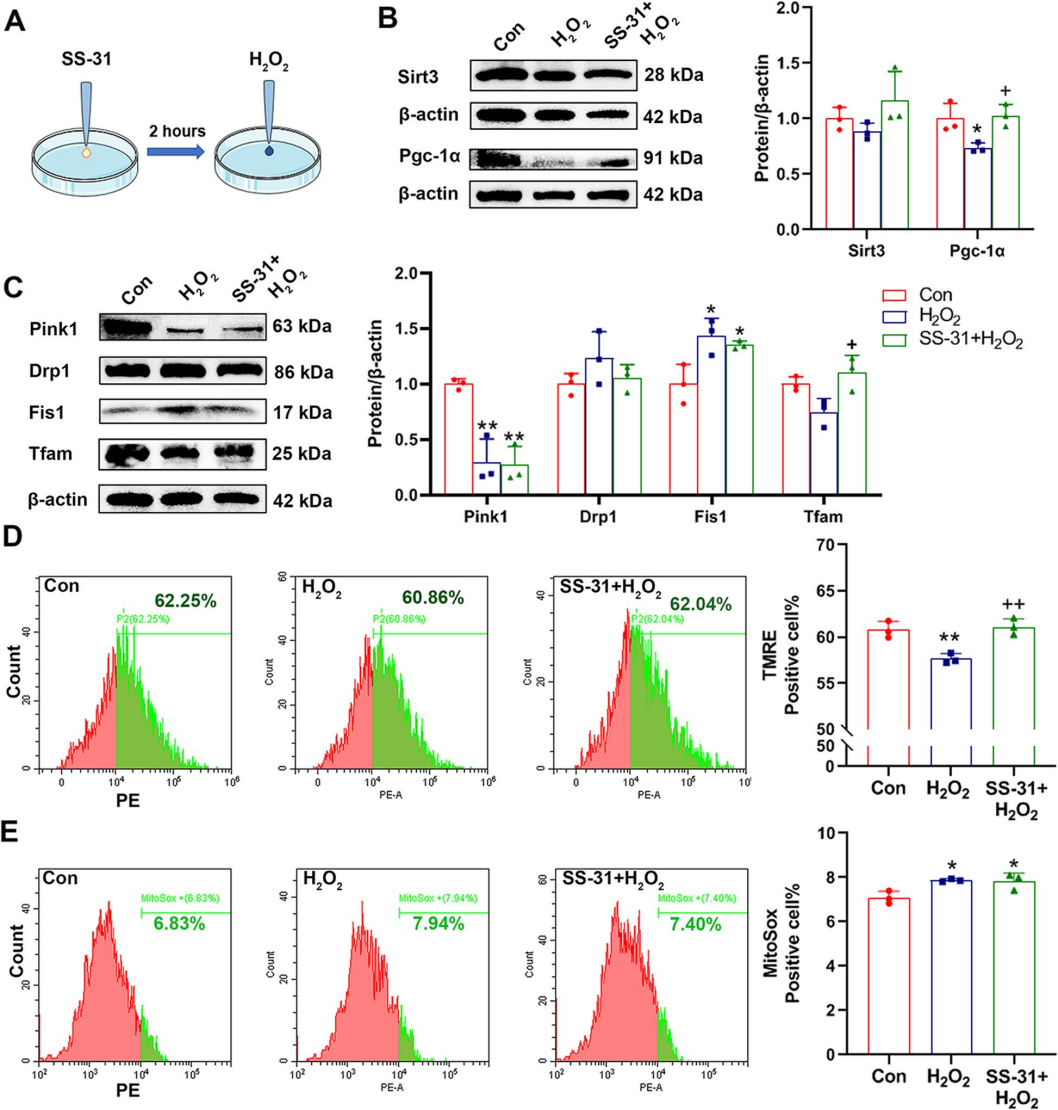
SS-31 restored mitochondrial function and mitochondrial homeostasis in H_2_O_2_-induced PC12 cells. **A** PC12 cells were treated with SS-31 (100 nM) for 2 h, followed by additional H_2_O_2_ (300 nM) for 12 h. **B** Western blot analysis of Sirt3 and Pgc-1α (F(2, 6) = 7.438, ^*^p = 0.0237; ^*^p = 0.0452, ^+^*p* = 0.0292). Protein levels of Sirt3 and Pgc-1α were quantified normalized to β-actin. n = 3 per group; One-way ANOVA. (C) Western blot analysis of Tfam (F(2, 6) = 6.981, ^*^p = 0.0272; ^+^*p* = 0.0254), Drp1, Fis1 (F(2, 6) = 8.292, ^*^p = 0.0188; ^*^p_(H2O2)_ = 0.0200, ^*^p_(SS-31+H2O2)_ = 0.0485) and Pink1 (F(2, 6) = 20.93, ^**^p = 0.0020; ^**^p_(H2O2)_ = 0.0037, ^**^p_(SS-31+H2O2)_ = 0.0030). Protein levels were quantified normalized to β-actin. *n* = 3 per group; One-way ANOVA. **D** and **E** Flow cytometric analysis was used to examine MMP (F(2, 6) = 16.90, ^**^p = 0.0034; ^++^*p* = 0.0045) and ROS (F(2, 6) = 7.564, ^*^p = 0.0229; ^*^p_(H2O2)_ = 0.0314, ^*^p_(SS-31+H2O2)_ = 0.0388) levels in different groups. *n* = 3 per group; One-way ANOVA. Data are represented as Mean ± SD; ^*^p < 0.05 and ^**^p < 0.01 as compared to con group. ^+^*p* < 0.05 and ^++^*p* < 0.01 as compared to H_2_O_2_ group

To further investigate the effects of SS-31 on mitochondria, a series of experiments were performed in PC12 cells. We examined the MMP and mitochondrial ROS levels by flow cytometric analysis. As shown in Fig. 4D-E, TMRE positive cell percent increased in H_2_O_2_ + SS-31 group compared to H_2_O_2_ group, but no significant difference in MitoSox positive cell percent, indicating SS-31 can improve mitochondrial function partially. Expression of Pgc-1α and Tfam increased in SS-31 + H_2_O_2_ group compared to H_2_O_2_ group, while Sirt3, Pink1, Drp1 and Fis1 protein level had no significant changes (Fig. 4B-C). Similarly, these results showed that SS-31 maintained mitochondrial homeostasis, particularly mitochondrial biogenesis.

Collectively, SS-31 restored mitochondrial function impaired by repeated IS infusion or H_2_O_2_ stimulation partially, and rebalanced mitochondrial homeostasis mainly through mitochondrial biogenesis. Moreover, SS-31 reversed IS-induced Sirt3 decline.

### Inhibition of Sirt3/Pgc-1α partially blocked the effect of SS-31 on headache treatment

**Fig. 5:**
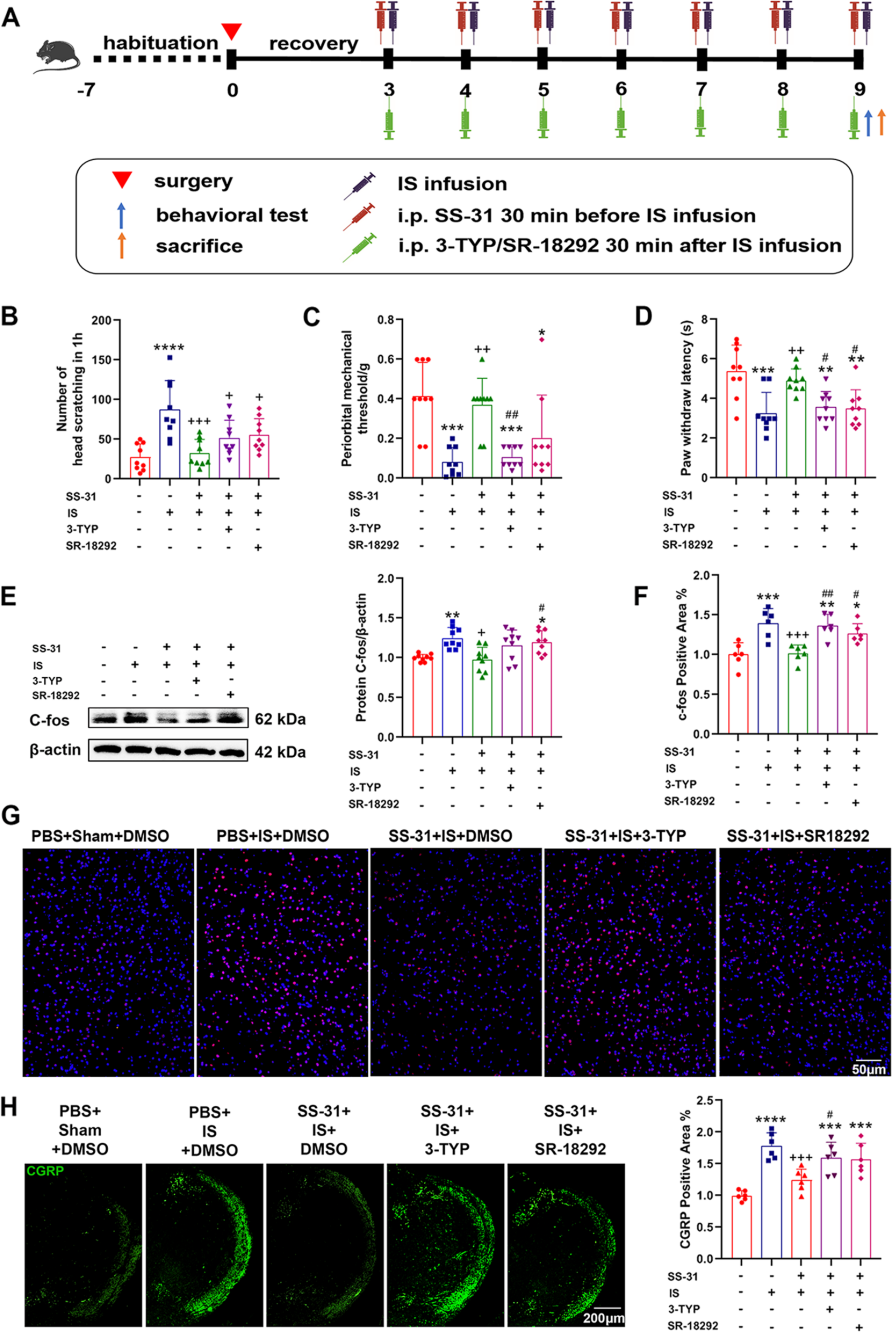
Inhibition of Sirt3/Pgc-1α partially blocked the effect of SS-31 on headache treatment. **A** Schematic diagram of the experiment. C57BL/6 mice received PBS + sham + DMSO, PBS + IS + DMSO, SS-31 + IS + DMSO, SS-31 + IS + 3-TYP or SS-31 + IS + SR-18292 treatment for 7 consecutive days, followed by behavioral tests and sacrifice to evaluate nociceptive responses. **B**-**D** The number of head scratching in 1 h (F(4, 40) = 9.107, ^****^p < 0.0001; ^****^p_(PBS+IS+DMSO)_ < 0.0001, ^+++^*p*_(SS-31+IS+DMSO)_ = 0.0001, ^+^*p*_(SS-31+IS+3-TYP)_ = 0.015, ^+^*p*_(SS-31+IS+SR-18292)_ = 0.0475, periorbital mechanical threshold (F(4, 40) = 9.986, ^****^p < 0.0001; ^***^p_(PBS+IS+DMSO)_ = 0.0001, ^***^p_(SS-31+IS+3-TYP)_ < 0.0004, ^*^p_(SS-31+IS+SR-18292)_ = 0.0260, ^++^*p*_(SS-31+IS+DMSO)_ = 0.0011, ^##^*p*_(SS-31+IS+3-TYP)_ = 0.0031) and paw withdrawal latency (F(4, 40) = 8.679, ^****^p < 0.0001; ^***^p_(PBS+IS+DMSO)_ = 0.0003, ^**^p_(SS-31+IS+3-TYP)_ = 0.0027, ^**^p_(SS-31+IS+SR-18292)_ = 0.0015, ^++^*p*_(SS-31+IS+DMSO)_ = 0.0080, ^#^*p*_(SS-31+IS+3-TYP)_ = 0.0464, ^#^*p*_(SS-31+IS+SR-18292)_ = 0.0288) were recorded in different groups. *n* = 9 per group; One-way ANOVA. **E**–**G** Western blot analysis (*n* = 9 per group; F(4, 40) = 6.389, ^***^p = 0.0004; ^**^p_(PBS+IS+DMSO)_ = 0.0058, ^*^p_(SS-31+IS+SR-18292)_ = 0.0451, ^++^*p*_(SS-31+IS+DMSO)_ = 0.0022, ^#^*p*_(SS-31+IS+SR-18292)_ = 0.0195) and immunofluorescence staining (*n* = 6 per group; F(4, 25) = 10.36, ^****^p < 0.0001; ^***^p_(PBS+IS+DMSO)_ = 0.0005, ^**^p_(SS-31+IS+3-TYP)_ = 0.0017, ^*^p_(SS-31+IS+SR-18292)_ = 0.0286, ^+++^*p*_(SS-31+IS+DMSO)_ = 0.0009, ^##^*p*_(SS-31+IS+3-TYP)_ = 0.0029, ^#^*p*_(SS-31+IS+SR-18292)_ = 0.0446) illustrated that expression levels of c-fos in TNC of different groups. Scale bar, 50 μm. One-way ANOVA. **H** Immunofluorescence staining (F(4, 25) = 14.31, ^****^p < 0.0001; ^****^p_(PBS+IS+DMSO)_ < 0.0001, ^***^p_(SS-31+IS+3-TYP)_ = 0.0003, ^****^p_(SS-31+IS+SR-18292)_ = 0.0004, ^+++^*p*_(SS-31+IS+DMSO)_ = 0.0009, ^#^*p*_(SS-31+IS+SR-18292)_ = 0.0425) was used to examine the levels of CGRP in TNC of different groups. Scale bar, 200 μm. *n* = 6 per group; One-way ANOVA. Data are represented as Mean ± SD; ^*^p < 0.05, ^**^p < 0.01 and ^***^p < 0.001 as compared to PBS + Sham + DMSO group. ^+^*p* < 0.05, ^++^*p* < 0.01 and ^+++^*p* < 0.001 as compared to PBS + IS + DMSO group. ^#^*p* < 0.05 as compared to SS-31 + IS + DMSO group

To further investigate whether Sirt3 and Pgc-1α are involved in the mechanisms of SS-31 on headache treatment, 3-TYP/SR-18292 was administered to inhibit the Sirt3/Pgc-1α after injection of SS-31 in an IS-induced mouse model. We recorded the number of head scratching in 1 h, as shown in Fig. 5B, which increased in SS-31 + IS + 3-TYP and SS-31 + IS + SR-18292 groups compared with SS-31 + IS + DMSO group without a significant difference. The periorbital mechanical threshold decreased significantly in the SS-31 + IS + 3-TYP group, while the paw withdraw latency decreased significantly in the SS-31 + IS + 3-TYP group and SS-31 + IS + SR-18292 group compared with the SS-31 + IS + DMSO group (Fig. 5C-D). Additionally, western blot and immunofluorescence analysis showed that the expression levels of c-fos and CGRP increased in SS-31 + IS + 3-TYP and SS-31 + IS + SR-18292 groups compared with the SS-31 + IS + DMSO group (Fig. 5E-H). These results indicated that the treatment effect of SS-31 can be depleted partially by 3-TYP/SR-18292.

### The effects of SS-31 on restoring mitochondrial function and maintaining mitochondrial homeostasis were partially counteracted by the inhibitor of Sirt3/Pgc-1α

**Fig. 6:**
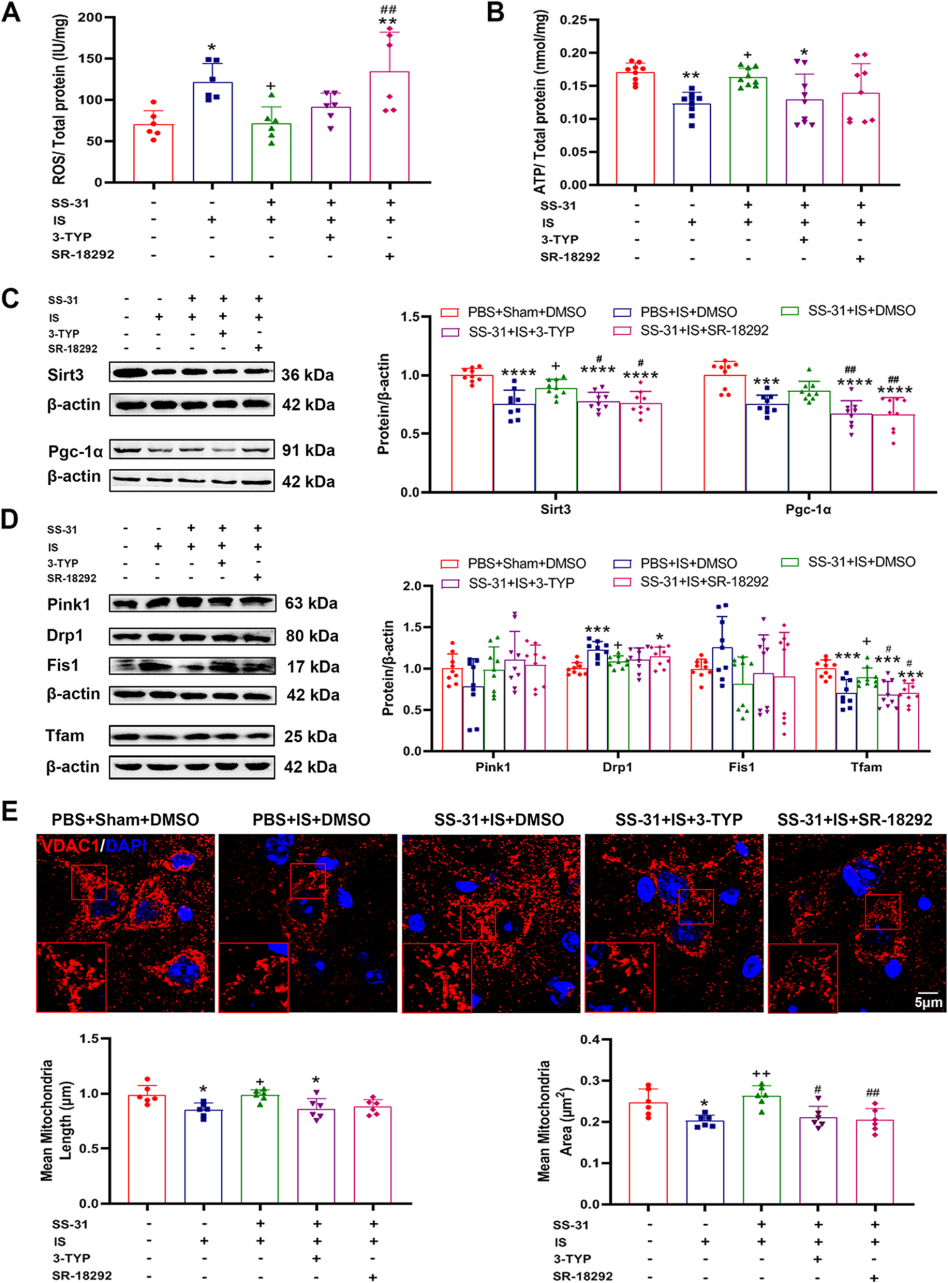
The effects of SS-31 on mitochondrial function and homeostasis were partially counteracted by inhibiting Sirt3/Pgc-1α. C57BL/6 mice received PBS + sham + DMSO, PBS + IS + DMSO, SS-31 + IS + DMSO, SS-31 + IS + 3-TYP or SS-31 + IS + SR-18292 treatment for 7 consecutive days, followed by sacrifice to evaluate mitochondrial function and mitochondrial homeostasis in TNC. **A** ROS levels were examined in different groups and normalized by total protein concentrations. *n* = 6 per group; One-way ANOVA. F(4, 25) = 7.078, ^***^p = 0.0006; ^*^p_(PBS+IS+DMSO)_ = 0.0222, ^**^p_(SS-31+IS+SR-18292)_ = 0.0029, ^+^*p*_(SS-31+IS+DMSO)_ = 0.0259, ^##^*p*_(SS-31+IS+SR-18292)_ = 0.0034. **B** ATP levels were examined in different groups and normalized by total protein concentrations. *n* = 9 per group; One-way ANOVA. F(4, 40) = 4.753, ^**^p = 0.0031; ^**^p_(PBS+IS+DMSO)_ = 0.0096, ^*^p_(SS-31+IS+3-TYP)_ = 0.0301, ^+^*p*_(SS-31+IS+DMSO)_ = 0.0393. **C** Western blot analysis showed that expression of Sirt3 (F(4, 40) = 13.71, ^****^p < 0.0001; ^****^p_(PBS+IS+DMSO)_ < 0.0001, ^****^p_(SS-31+IS+3-TYP)_ < 0.0001, ^****^p_(SS-31+IS+SR-18292)_ < 0.0001, ^+^*p*_(SS-31+IS+DMSO)_ = 0.0153, ^##^*p*_(SS-31+IS+3-TYP)_ = 0.0487, ^##^*p*_(SS-31+IS+SR-18292)_ = 0.0216) and Pgc-1α (F(4, 40) = 15.15, ^****^p < 0.0001; ^***^p_(PBS+IS+DMSO)_ = 0.0002, ^****^p_(SS-31+IS+3-TYP)_ < 0.0001, ^****^p_(SS-31+IS+SR-18292)_ < 0.0001, ^##^*p*_(SS-31+IS+3-TYP)_ = 0.0044, ^##^*p*_(SS-31+IS+SR-18292)_ = 0.0039) in different groups. *n* = 9 per group; One-way ANOVA. (D) Representative immunoblots and quantification illustrated the levels of Tfam (F(4, 40) = 9.868, ^****^p < 0.0001; ^***^p_(PBS+IS+DMSO)_ = 0.0004, ^***^p_(SS-31+IS+3-TYP)_ = 0.0001, ^***^p_(SS-31+IS+SR-18292)_ = 0.0003, ^+^*p*_(SS-31+IS+DMSO)_ = 0.0417, ^#^*p*_(SS-31+IS+3-TYP)_ = 0.0177, ^#^*p*_(SS-31+IS+SR-18292)_ = 0.0363), Drp1 (F(4, 40) = 5.655, ^**^p = 0.0011; ^***^p_(PBS+IS+DMSO)_ = 0.0004, ^*^p_(SS-31+IS+SR-18292)_ = 0.0492, ^+^*p*_(SS-31+IS+DMSO)_ = 0.0429), Fis1 and Pink1 in different groups. *n* = 9 per group; One-way ANOVA. **E** Immunofluorescence staining of VDAC1 and nucleus (DAPI) in TNC. The mean mitochondria length (F(4, 25) = 5.225, ^**^p = 0.0034; ^*^p_(PBS+IS+DMSO)_ = 0.0232, ^*^p_(SS-31+IS+3-TYP)_ = 0.0410, ^+^*p*_(SS-31+IS+DMSO)_ = 0.0312) and area (F(4, 25) = 6.964, ^***^p = 0.0007; ^*^p_(PBS+IS+DMSO)_ = 0.0396, ^++^*p*_(SS-31+IS+DMSO)_ = 0.0033, ^#^*p*_(SS-31+IS+3-TYP)_ = 0.0150, ^##^*p*_(SS-31+IS+SR-18292)_ = 0.0047) were calculated. Scale bar, 5 μm. *n* = 6 per group; One-way ANOVA. Data are represented as Mean ± SD; ^*^p < 0.05, ^**^p < 0.01, ^***^p < 0.001 and ^****^p < 0.0001 as compared to PBS + Sham + DMSO group. ^+^*p* < 0.05 and ^++^*p* < 0.01 as compared to PBS + IS + DMSO group. ^#^*p* < 0.05 and ^##^*p* < 0.01 as compared to SS-31 + IS + DMSO group

We further assessed mitochondrial function and mitochondrial homeostasis after administration of Sirt3/Pgc-1α inhibitors. The ROS level increased markedly in SS-31 + IS + SR-18292 group compared to the SS-31 + IS + DMSO group, but there was no significant difference between SS-31 + IS + 3-TYP and SS-31 + IS + DMSO groups (Fig. 6A). The ATP levels of SS-31 + IS + 3-TYP and SS-31 + IS + SR-18292 groups decreased compared to the SS-31 + IS + DMSO group with no significant difference (Fig. 6B). Then, our findings showed that the expression levels of Sirt3 and Pgc-1α decreased in SS-31 + IS + 3-TYP and SS-31 + IS + SR-18292 groups compared to SS-31 + IS + DMSO group (Fig. 6C), revealing that the expression of Sirt3 and Pgc-1α decreased by the inhibitor of each other. Tfam expression decreased significantly in SS-31 + IS + 3-TYP and SS-31 + IS + SR-18292 groups compared to SS-31 + IS + DMSO group (Fig. 6D). Furthermore, immunofluorescence staining showed that there were more fragmented mitochondria in SS-31 + IS + 3-TYP group and SS-31 + IS + SR-18292 group compared to SS-31 + IS + DMSO group (Fig. 6E). The mean mitochondria areas in the SS-31 + IS + 3-TYP group and SS-31 + IS + SR-18292 group were markedly lower than the SS-31 + IS + DMSO group.

Altogether, our findings suggested that the effect of SS-31 on restoring mitochondrial function and mitochondrial homeostasis, especially mitochondrial biogenesis, was partially counteracted by the inhibitor of Sirt3/Pgc-1α. In addition, these results revealed possible links between Sirt3 and Pgc-1α.

### Overexpression of Sirt3/Pgc-1α enhanced expression of each other and improved mitochondrial function in PC12 cells

**Fig. 7:**
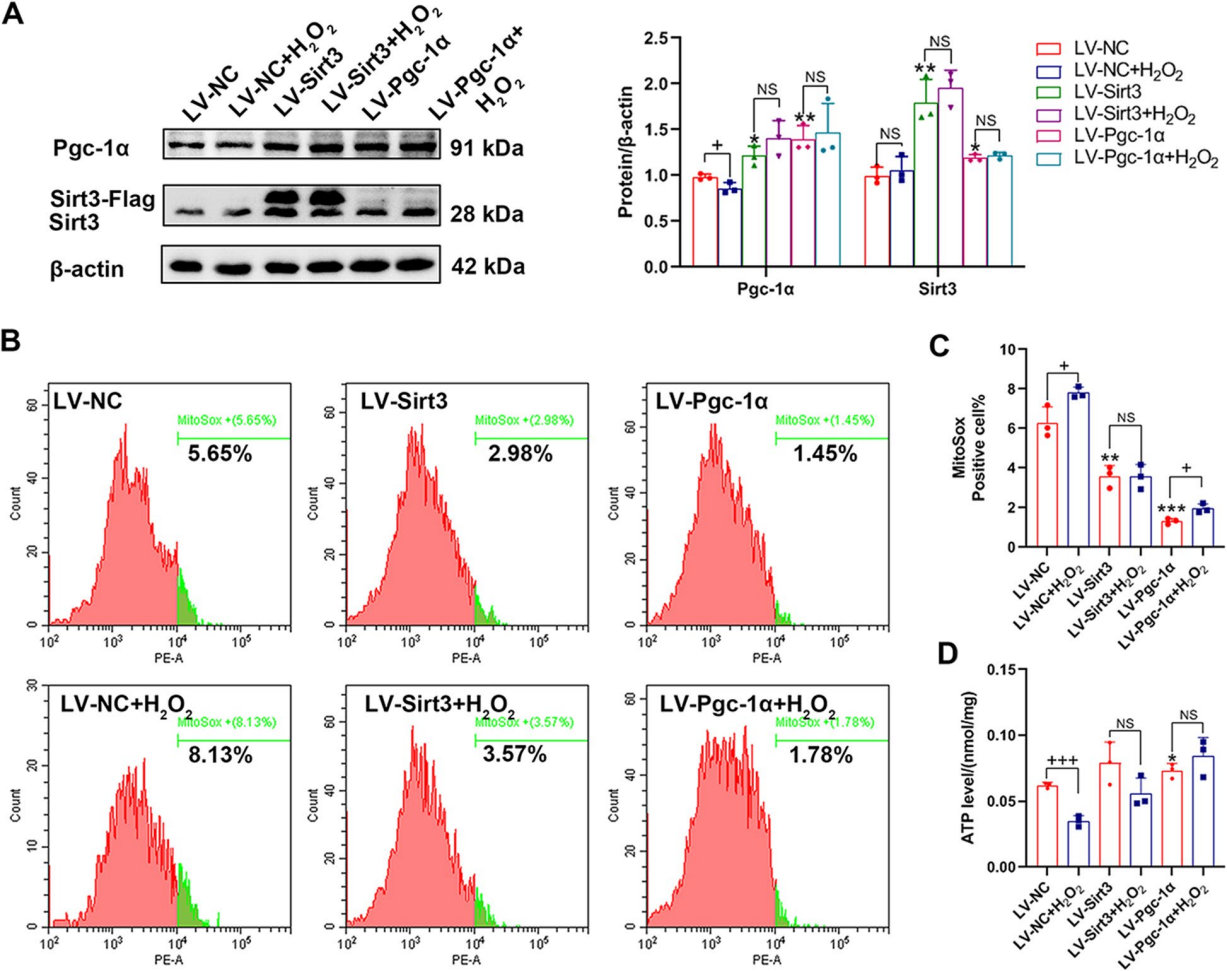
Overexpression of Sirt3/Pgc-1α enhanced expression of each other and improved mitochondrial function in PC12 cells. PC12 cells infected by lentivirus (LV) were treated with/without H_2_O_2_ (300 nM) for 12 h. **A** Western blot analysis of Sirt3 (^**^p_(LV-Sirt3)_ = 0.0066, ^*^p_(LV-Pgc1α)_ = 0.0311) and Pgc-1α (^+^*p* = 0.05; ^*^p_(LV-Sirt3)_ = 0.0242, ^**^p_*(*LV-Pgc1α)_ = 0.0096). *n* = 3 per group; Student’s* t*-test. **B**-**C** Flow cytometric analysis was used to detected the ROS levels in different groups. n = 3 per group; Student’s* t*-test. ^+^*p*_(LV-NC)_ = 0.0371, ^+^*p*_(LV-Pgc1α)_ = 0.0158; ^**^p_(LV-Sirt3)_ = 0.0019, ^***^p_(LV-Pgc1α)_ = 0.0001. **D** The ATP levels were examined and normalized by total protein concentrations in different groups. *n* = 3 per group; Student’s* t*-test. ^+++^*p*_(LV-NC)_ = 0.0005; ^*^p_(LV-Pgc1α)_ = 0.0295. Data are represented as Mean ± SD; ^*^p < 0.05, ^**^p < 0.01 and ^***^p < 0.001 as compared to LV-NC group. ^+^*p* < 0.05 and ^+++^*p* < 0.001 as compared to LV- group. NC, negative control. NS, no significance

To further explore whether Sirt3/Pgc-1α positive feedback loop existed, Sirt3 and Pgc-1α were overexpressed in PC12 cells, respectively. At the same time, we examined the effect of Sirt3 and Pgc-1α on mitochondrial function under H_2_O_2_–induced oxidative stress. We found that when Sirt3 was overexpressed, the expression level of Pgc-1α increased significantly compared to LV-negative control (NC) group. Similarly, when Pgc-1α was overexpressed, the expression level of Sirt3 increased with a significant difference from LV-NC group (Fig. 7A). Combined with the above-discussed results of Fig. 6C, a positive feedback loop between Sirt3 and Pgc-1α existed.

After treatment with H_2_O_2_, the protein level of Pgc-1α decreased significantly in LV-NC + H_2_O_2_ group compared to LV-NC group, while the level of Sirt3 had no change. As expected, expressions of Sirt3 and Pgc-1α were no significant difference between LV-Sirt3 and LV-Sirt3 + H_2_O_2_ group, identically between LV-Pgc-1α and LV-Pgc-1α + H_2_O_2_ group (Fig. 7A). Flow cytometric analysis showed that MitoSox positive cell percent in LV-Sirt3 and LV-Pgc-1α group were decreased remarkably in LV-NC + H_2_O_2_ group compared to LV-NC group (Fig. 7B-C). In addition, we found that the ATP levels of LV-NC + H_2_O_2_ group decreased compared to the LV-NC group, while the ATP levels of LV-Sirt3 and LV-Sirt3 + H_2_O_2_ were no difference, identically between LV-Pgc-1α and LV-Pgc-1α + H_2_O_2_ groups (Fig. 7D). These results indicated that Sirt3 and Pgc-1α participated in regulating mitochondrial function.

### No interaction of Sirt3 and Pgc-1α complexes was detected

**Fig. 8:**
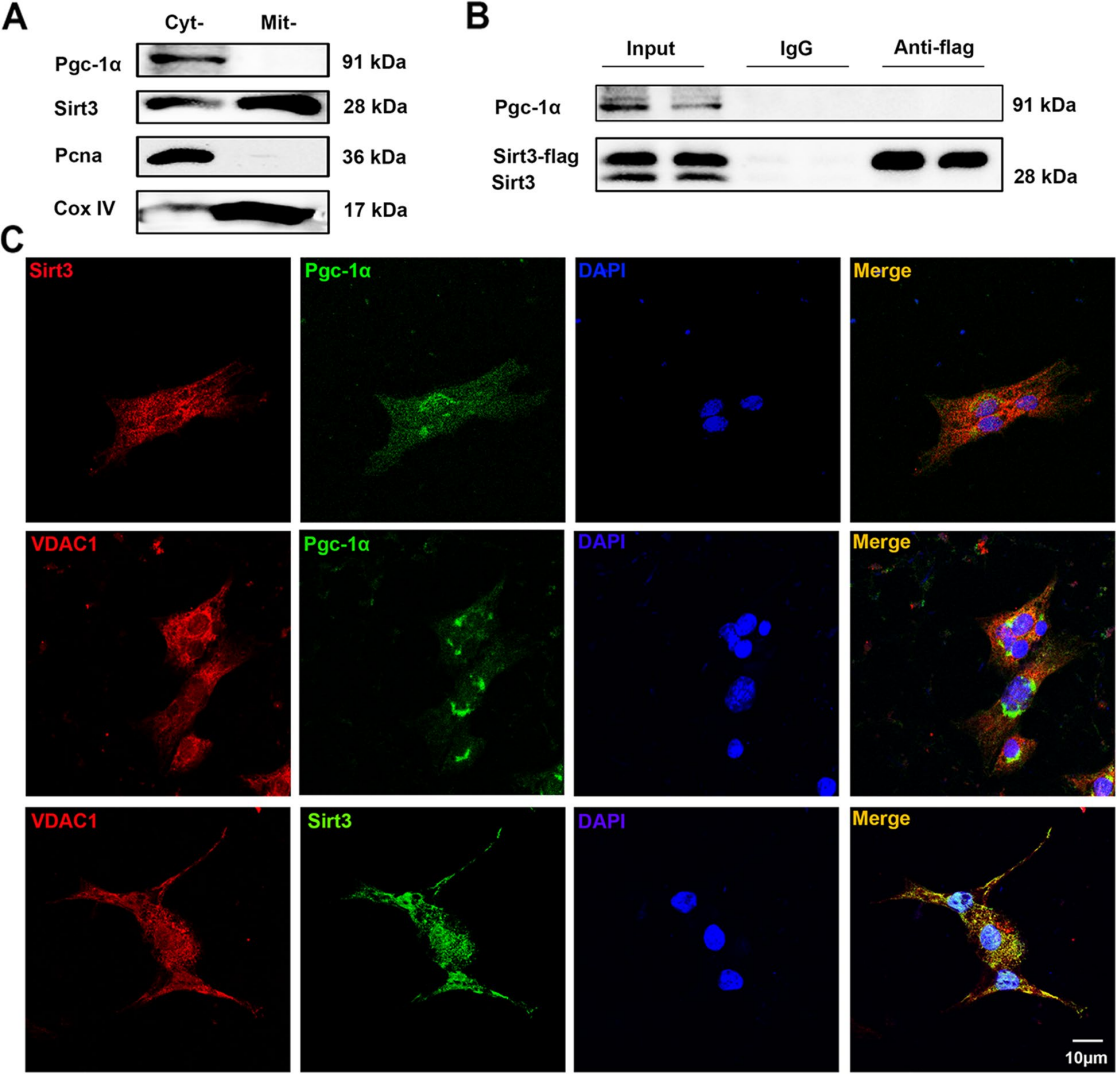
No interaction of Sirt3 and Pgc-1α complexes was detected in PC12 cells. Wild-type PC12 cells were cultured for western blot analysis and immunofluorescence staining, while PC12 cells infected by LV-Sirt3 were cultured for co-IP analysis. **A** Western blot analysis of Sirt3 and Pgc-1α to evaluate sub-cellular localization. **B** Co-IP analysis of Sirt3 and Pgc-1α. **C** Immunofluorescence staining of Sirt3, Pgc-1α and VDAC1. Scale bar, 10 μm. Cyt-: cytoplasm; Mit-: mitochondria

Finally, we explored how the Sirt3/Pgc-1α positive feedback loop formed. Some studies revealed that Sirt3 could regulate the expression of Pgc-1α [14, 22], but it was unclear whether there was an interaction between Sirt3 and Pgc-1α. We examined the locations of Sirt3 and Pgc-1α by western blot and immunofluorescence analysis. In PC12 cells, mitochondrial and cytosolic proteins were isolated by a mitochondria isolation kit. Cox IV and Pcna were respectively used to exclude interference of mitochondria and nuclei. We found that Sirt3 was mainly located in mitochondria while Pgc-1α was located in nucleus and cytoplasm, and colocalization between the two was rarely detected (Fig. 8A and C). Then, co-IP results revealed that there was no obvious interaction between Sirt3 and Pgc-1α (Fig. 8B). We obtained the similar results in 293 T cells by co-IP and immunofluorescence analysis (Supplementary Fig. 3). These results indicated that there was no interaction between the two proteins. Further experiments are necessary to explore how Sirt3/Pgc-1α positive feedback loop forms.

## Discussion

In this study, we used an IS-induced headache rodent model to investigate whether SS-31 had a therapeutic effect on headache and the possible mechanisms involved. Our results revealed that mitochondrial dysfunction and mitochondrial homeostasis imbalance occurred after repeated IS infusion. SS-31 alleviated IS-induced nociceptive responses, restored mitochondrial function and mitigated mitochondrial homeostasis imbalance. The treatment of SS-31 for headache were partially blocked by inhibitor of Sirt3/Pgc-1α. Overexpression of Sirt3 or Pgc-1α increased the protein level of each other, indicating that a Sirt3/Pgc-1α positive feedback loop formed. However, no protein–protein interaction of Sirt3 and Pgc-1α was observed. We concluded that SS-31 alleviated nociceptive responses and restored mitochondrial function in IS-induced headache via Sirt3/Pgc-1α positive feedback loop.

Increasing evidence indicated that migraine is a syndrome with mitochondrial dysfunction [5–7]. Clinically, several antioxidant drugs have been proven to have therapeutic effects on migraine prevention, including vitamin and coenzyme Q_10_ [7]. SS-31, as a novel antioxidant, has unique superiorities compared to these drugs: (1) As a mitochondrial targeting tetrapeptide, SS-31 has no effects on normal mitochondria and is clinically relatively safe [37, 38]. (2) SS-31 is imported into mitochondria independent of the MMP [39]. Owing to impaired MMP under pathological conditions, the efficacy of mitochondria-targeted antioxidants which rely on MMP may be attenuated [40]. Thus, we suggest that SS-31 has the potential to be an effective drug candidate for migraine treatment.

Several commonly used migraine animal models, such as the nitroglycerine model and the cortical spreading depression model, all have their own disadvantages and cannot model all clinical features of migraine [41, 42]. The nitroglycerine model, as the most commonly used migraine model, is not suitable for a study about mitochondrial function because of the detrimental effects of nitroglycerine on mitochondria, including increased ROS production, Ca^2+^ accumulation and oxidative stress [43, 44]. IS-induced headache model has been successfully used to investigate the mechanisms of meningeal and trigeminovascular nociception, and can model some features that are believed to be associated strongly with migraine, such as recurrent headache and repeated activation of the trigeminovascular system [45]. Therefore, we chose the IS-induced headache model in our study. PC12 cell line, which has some neuron properties of neuron cells, is widely used in in vitro studies of the pathological and physiological process of neuron cells. The PC12 cell model induced by H_2_O_2_ is the most commonly used cell model for investigating the molecular mechanisms of some candidate drugs with protective effects on neuron cells [46] and mitochondrial dysfunction. Therefore, the PC12 cell line was used to investigate the treatment of SS-31 on mitochondria and the underlying mechanisms in our work.

Firstly, we explored whether mitochondrial dysfunction and mitochondrial homeostasis imbalance occurred in an IS-induced headache mouse model. Our findings showed that accumulation of lipid hydroperoxides and ROS and inhibition of energy production occurred in the TNC after repeated IS infusion (Fig. 1D). Consistent with our results, a study reported that the ATP content and MMP level decreased significantly in the TNC of IS-induced rat models [10]. Mitochondrial homeostasis is a process to maintain mitochondrial function. A recent study showed that in trigeminal ganglion (TG) neurons, mitochondrial biogenesis was inhibited and mitochondrial dynamics was shifted towards fission in IS-induced rat models [13]. Consistently, we found that mitochondrial biogenesis and mitophagy were suppressed, while the mitochondrial fission increased in TNC after repeated IS infusion (Fig. 1A-C).

We further explored whether SS-31 had a therapeutic effect on headache by detecting the nociceptive responses in an IS-induced mouse model, including migraine-like behavior, hyperalgesia, the release of CGRP and activation of nociceptive neurons (Fig. 2). We found that SS-31 apparently reversed IS-induced nociceptive responses. Results from numerous studies have shown that SS-31 restored mitochondrial function [30, 47] and maintained mitochondrial homeostasis by enhancing mitochondrial biogenesis and mitophagy, rebalancing mitochondrial dynamics in a variety of human diseases [39, 48]. Consistent with these studies, our results showed that mitochondrial dysfunction induced by repeated IS infusion was reversed and mitochondrial biogenesis was enhanced significantly by SS-31 (Fig. 3). However, the levels of Drp1 and P62 had no significant difference between the sham group and IS group, which was incompatible with the results in Fig. 1. A small sample size probably is responsible for the inconsistency.

Sirt3 has been proven to play essential roles in the maintenance of mitochondrial function and metabolism in human diseases [14–16]. It’s confirmed that upregulating Sirt1, another Sirt family member, alleviated mitochondrial dysfunction in TNC of migraine rat models [10, 49]. However, until now, no study has explored the role of Sirt3 on migraine. Based on the proven fact that Sirt3 and Pgc-1α take part in maintaining mitochondrial homeostasis [14–18, 50], we investigated whether Sirt3 and Pgc-1α play roles in the effects of SS-31 on IS-induced headache. Our findings showed that the inhibitors of Sirt3 and Pgc-1α increased nociceptive responses, injured mitochondrial function and suppressed mitochondrial biogenesis (Figs. 5 and 6), indicating that Sirt3 and Pgc-1α may represent potential targets for therapeutic intervention of headache.

It is noteworthy that Sirt3 and Pgc-1α expressions were downregulated by each other’s inhibitor in Fig. 6C. As reported, Sirt3 promoted Pgc-1α expression by impacting AMPK (a protein kinase that directly phosphorylates and activates Pgc-1α) and CREB (a transcription factor that promotes Pgc-1α expression) phosphorylation [15, 23, 51]. Notably, Sirt3 is a downstream molecule of Pgc-1α [23]. Consistent with these findings, our results indicated that overexpression of Sirt3 and Pgc-1α promoted the level of each other (Fig. 7A). Combined with our result in Fig. 6C, it is proven that Sirt3 and Pgc-1α can form a positive feedback loop. However, whether a protein–protein interaction exists between Sirt3 and Pgc-1α remains unclear. As reported, Sirt3 was mainly localized in the mitochondria, although a small number of studies found active Sirt3 outside of mitochondria [23, 52, 53]. Pgc-1α was mainly localized in nuclei, while a study reported that a Pgc-1α isoform was localized in mitochondria [23, 54]. Consistent with most of the studies, our study showed that Pgc-1α was located in nuclei and cytoplasm, while Sirt3 was mainly located in mitochondria. In addition, no obvious colocalization between the two was detected (Fig. 8). Further explorations are necessary to find out how Sirt3 and Pgc-1α promote the expression of each other.

Migraine is a female-predominant disorder. It is worth noting that a sexual dimorphism of mitochondria has been reported in many studies and the results showed that the mitochondrial function of female was stronger than male in the brain of both rodent animals [55–57] and human [58], which seems to do not explain the high incidence of women in migraine. There are two hypotheses to explain the sex differences in mitochondria: (1) Mitochondria are exclusively maternally inherited. (2) Sex hormones affect the mitochondria [59, 60]. However, a study reported that both mitochondrial variants and haplogroups had no association with migraine [61]. Consistently, another study found that there was no evidence of association of the studied mtSNP and haplogroups with migraine [62]. Therefore, maternal inheritance of mitochondria may not be a driver of frequent occurrence in women with migraine. In addition, the results in Supplementary Fig. 5 also indicated that sex-differences did not play a major role in our study. Sex steroids are considered to be a critical factor of the sex difference in migraine. To avoid the effects of cyclic variation in female hormonal levels on migraine, we mainly used male animals in our work. Notably, it is interesting and necessary to deeply investigate the association among sex, mitochondria and migraine in the future.

There are still several limitations in our study: (1) We used the repeated IS-induced headache animal model in our work. It is worthy to find out whether mitochondrial dysfunction occurs in other migraine models in further work. (2) Mainly using male animals in a study of migraine, a female-predominant disorder, is lacking in rigor. (3) Our study only showed the therapeutic effects of SS-31 on headache and mitochondrial dysfunction, but didn’t elucidate the causal relationship between headache and mitochondrial dysfunction. (4) There is still no mature and reliable cell model for migraine. In this study, we used a cell model of oxidative stress induced by H_2_O_2_ only to investigate the treatment of SS-31 for mitochondrial dysfunction and mitochondrial homeostasis rather than migraine. (5) The mechanisms of Sirt3/Pgc-1α positive feedback loop require further explorations.

## Conclusions

In conclusion, this study mainly investigated whether SS-31 has a therapeutic effect on headache and the possible mechanisms involved. SS-31 alleviated IS-induced nociceptive responses, mitochondrial dysfunction and mitochondrial homeostasis imbalance. These effects of SS-31 were partially blocked by inhibiting Sirt3 and Pgc-1α respectively, which forms a positive feedback loop. Our results have identified that SS-31 has the potential to be an effective drug candidate for headache treatment, and Sirt3/Pgc-1α positive feedback loop may represents an underlying target for therapeutic intervention of headache.

## Supplementary Information

**Additional file 1.**

## Data Availability

All data generated or analyzed during this study are included in this article (and its Additional file).

## Abbreviations

SS-31: Szeto-Schiller peptide
IS: Inflammatory soup
TNC: Trigeminal nucleus caudalis
ADP: Adenosine diphosphate
ATP: Adenosine triphosphate
Pgc-1α: Peroxisome proliferator-activated receptor-gamma co-activator 1α
Sirt3: Sirtuin 3
MMP: Mitochondrial transmembrane potential
IACUC: Institutional Animal Care and Use Committee
i.p.: Intraperitoneal injection
NGF: Nerve growth factor
LV: Lentivirus
PCR: Polymerase chain reaction
PMSF: Phenylmethylsulfonyl fluoride
BCA: Bicinchonic acid
PVDF: Polyvinylidene-difluoride
Tfam: Mitochondrial transcription factor A
Mfn2: Mitofusin 2
Fis1: Fission 1
Drp1: Dynamin related protein 1
Pink: PTEN-induced putative kinase 1
Pcna: Proliferating cell nuclear antigen
CoxIV: Cytochrome oxidase subunit IV
HRP: Horseradish peroxidase
RT: Room temperature
BSA: Bovine serum albumin
VDAC1: Voltage-dependent anion channel 1
CGRP: Calcitonin gene-related peptide
co-IP: Coimmunoprecipitation
TEM: Transmission electron microscope
ROS: Reactive oxygen species
ELISA: Enzyme linked immunosorbent assay
MDA: Malondialdehyde
TMRE: Tetramethylrhodamine ethyl ester perchlorate
Opa1: Optic atrophy 1
