# Supplementary material for: SS-31 alleviated nociceptive responses and restored mitochondrial function in a headache mouse model via Sirt3/Pgc-1α positive feedback loop
Source: J Headache Pain. 2023 Jun 5;24(1):65. doi: 10.1186/s10194-023-01600-6 (PMC10240765; doi:10.1186/s10194-023-01600-6)
Supplement: Supplementary file 1 — Additional file 1. [file 10194_2023_1600_MOESM1_ESM.docx]

**Supplementary Methods**

293T cells (gifted by Dr R.M. Zhou) were cultured in complete DMEM medium containing 10% fetal bovine serum and 1% penicillin/streptomycin. All cells were incubated at 37 °C in a 5% CO_2_ incubator. The cells between 10^th^ and 20^th^ generations were seeded in plates or dishes coated with polylysine (0.1 mg/mL) and cultured in complete medium for co-IP analysis and immunofluorescence staining.

**Supplementary Results**

To explore the effects of SS-31 alone, the behavioral tests were performed. Our results showed that the number of head scratching in 1 hour, the periorbital mechanical threshold and paw withdrawal latency all had no difference between the PBS+sham group and the SS-31+sham group (Supplementary Fig. 4A), suggesting that the drug SS-31 alone had no effect on behavior of mice received sham treatment. In addition, the western blot analysis showed that the protein levels of Pgc-1α, Tfam, Mfn2, Drp1, Fis1, P62 and Pink1 did not change after the last SS-31 injection in the SS-31+sham group compared to the PBS+sham group (Supplementary Fig. 4B-D). Our findings indicated that SS-31 alone had no effect on behavioral and mitochondrial homeostasis of mice received sham treatment.

**Supplementary Figures**

**
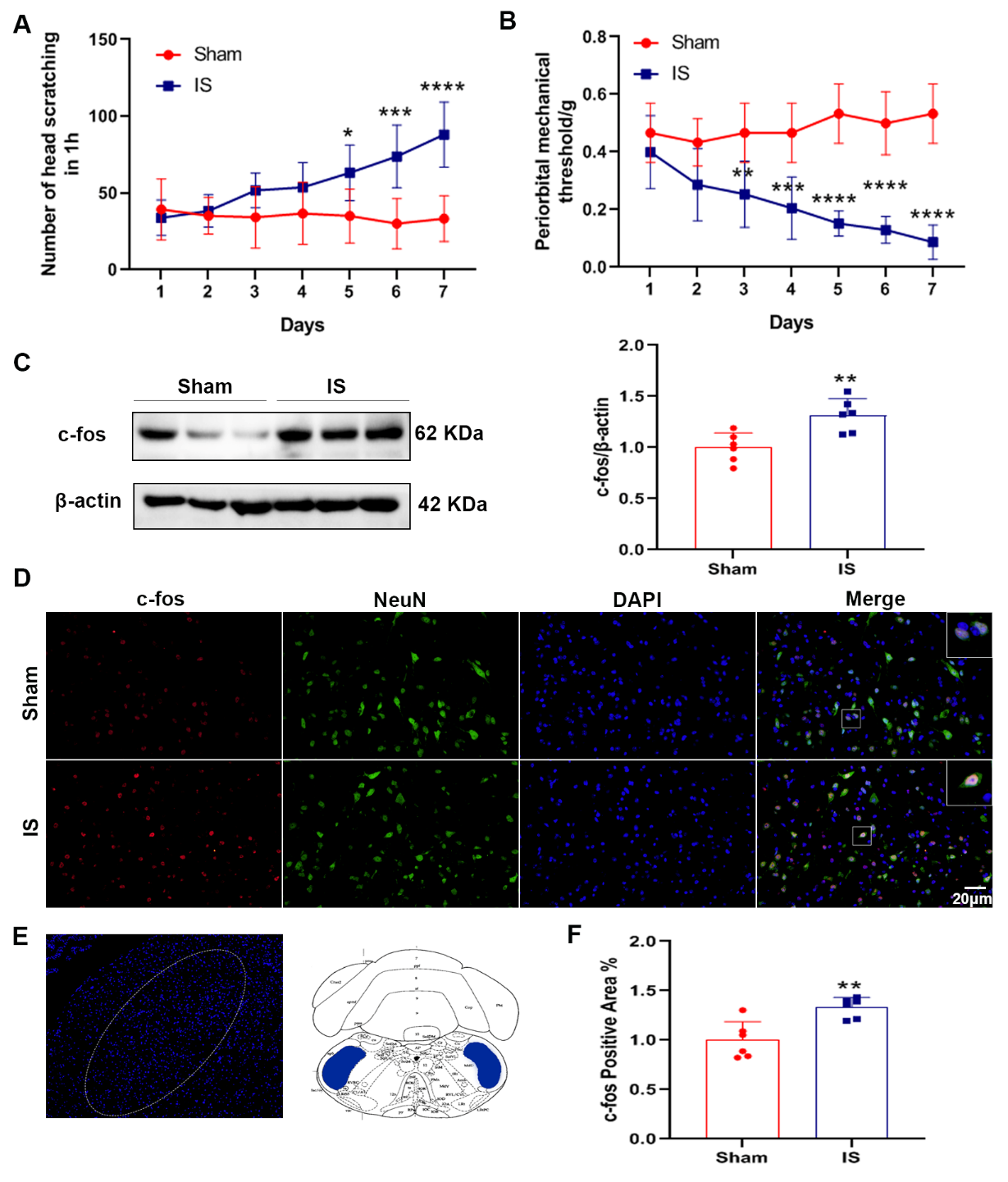
**

**Supplementary Fig. 1** Repeated IS infusion induced nociceptive responses and mitochondrial dysfunction. Male C57BL/6 mice were sham treated or dural-infused of inflammatory soup (IS) for 7 consecutive days, and then sacrificed to assess nociceptive responses. (A) The number of head scratching in 1 hour were recorded for 7 consecutive days. n=6 per group; Two-way ANOVA. Time F(1, 70)=38.25, ^****^*p*<0.0001; Treatment F(6, 70)=3.141, ^***^*p*=0.0087; ^*^*p*_(5d)_=0.0342, ^***^*p*_(6d)_=0.0002, ^****^*p*_(7d)_<0.0001. (B) The periorbital mechanical threshold was recorded for 7 consecutive days. n=6 per group; Two-way ANOVA. Time F(1, 70)=156.9, ^****^*p* <0.0001; Treatment F(6, 70)=2.102, ^***^*p*=0.0638; ^**^*p*_(3d)_= 0.0025, ^****^*p*_(4d)_=0.0001, ^****^*p*_(5d)_<0.0001, ^****^*p*_(6d)_<0.0001, ^****^*p*_(7d)_<0.0001. (C) Representative immunoblots and quantification of the protein levels of c-fos. n=6 per group; Student’s t-test. ^**^*p*=0.0050. (D and F) Immunofluorescence staining of c-fos and nucleus (DAPI) in the TNC. Scale bar, 50 μm. n=6 per group; Student’s *t*-test. ^**^*p*=0.0033. (E) The TNC (blue areas) was shown. Data are represented as Mean ± SD; ^*^*p*<0.05, ^**^*p*<0.01, ^***^*p*<0.001 and ^****^*p*<0.0001 as compared to sham group.

**
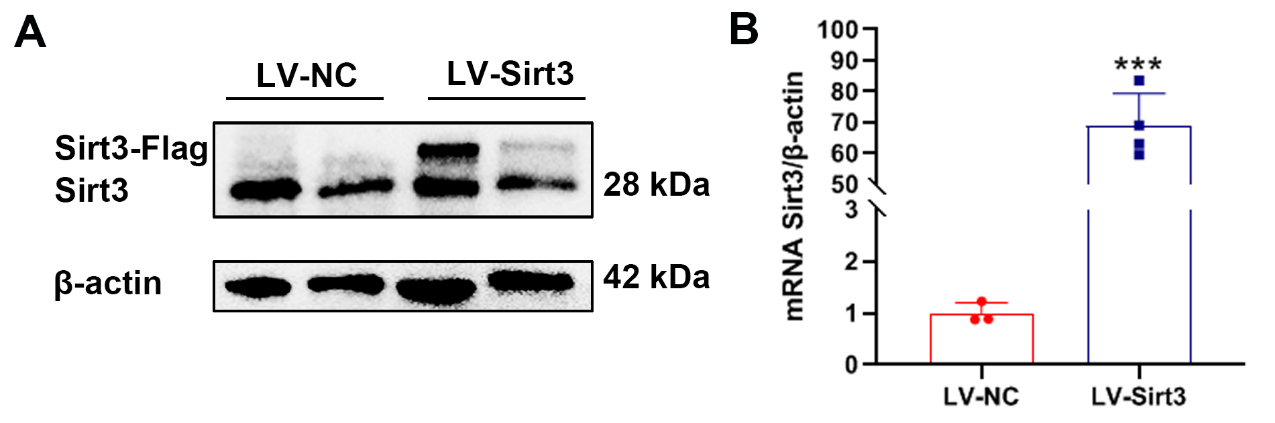
Supplementary Fig. 2** Validation of Sirt3 overexpression in PC12 cells. PC12 cells infected by lentivirus (LV) were collected to evaluate the mRNA and protein levels of Sirt3. (A) Representative immunoblots of Sirt3 expression in LV-NC and LV-Sirt3 groups. (B) RT-PCR showed that the mRNA levels of Sirt3 (^***^*p*=0.0001) in LV-NC and LV-Sirt3 groups. Student’s *t*-test was performed for statistical analysis. Data are represented as Mean ± SD; n=3/4 per group; ^***^*p*<0.001 as compared to LV-NC group. NC, negative control.


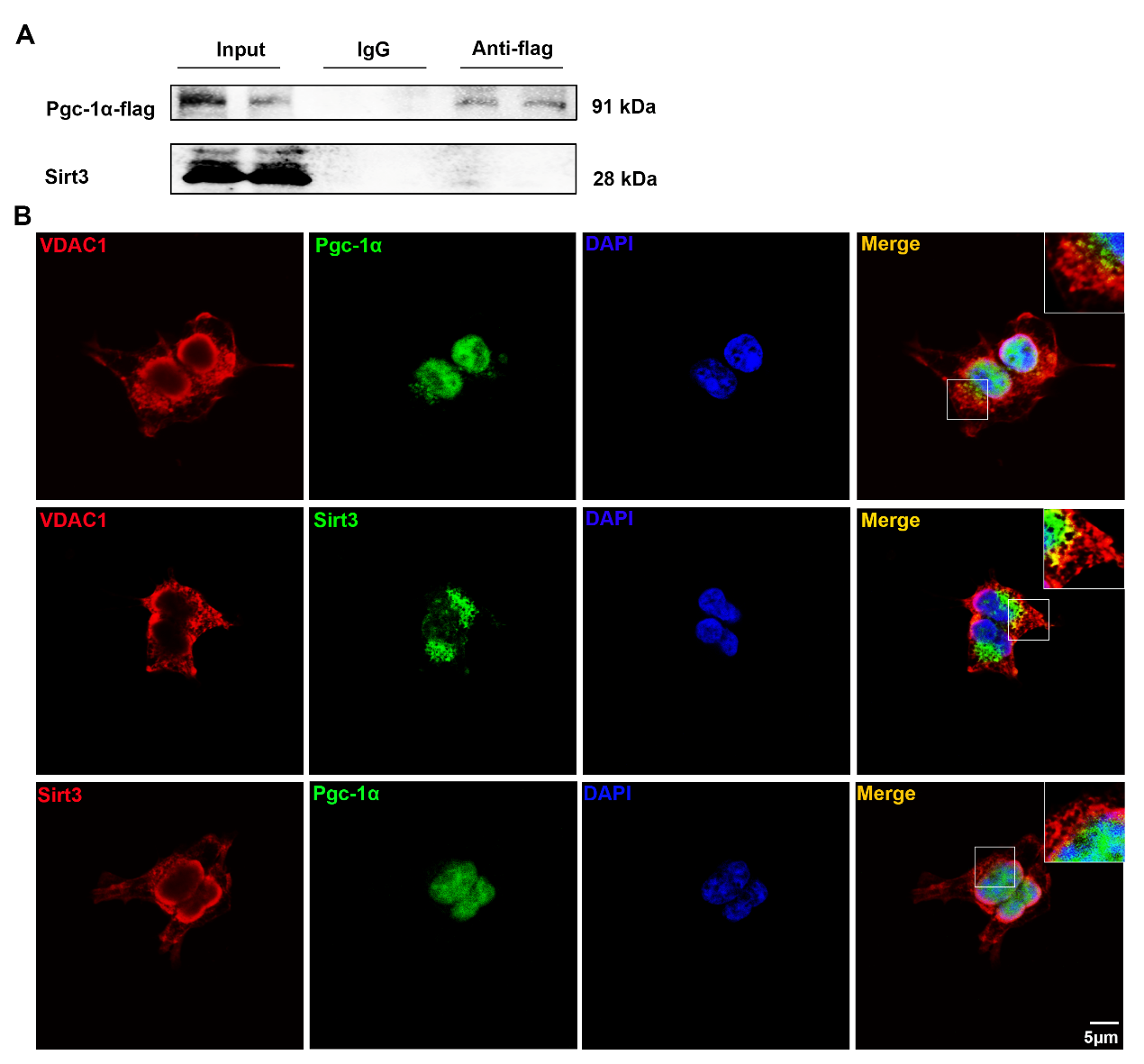


**Supplementary Fig. 3** No interaction of Sirt3 and Pgc-1α complexes was detected in 293T cells. Wild-type 293T cells were cultured for immunofluorescence staining, while 293T cells infected by LV-Pgc-1α were cultured for co-IP analysis. (A) Co-IP analysis of Sirt3 and Pgc-1α. (B) Immunofluorescence staining of Sirt3, Pgc-1α and VDAC1. Scale bar, 5 μm.

**
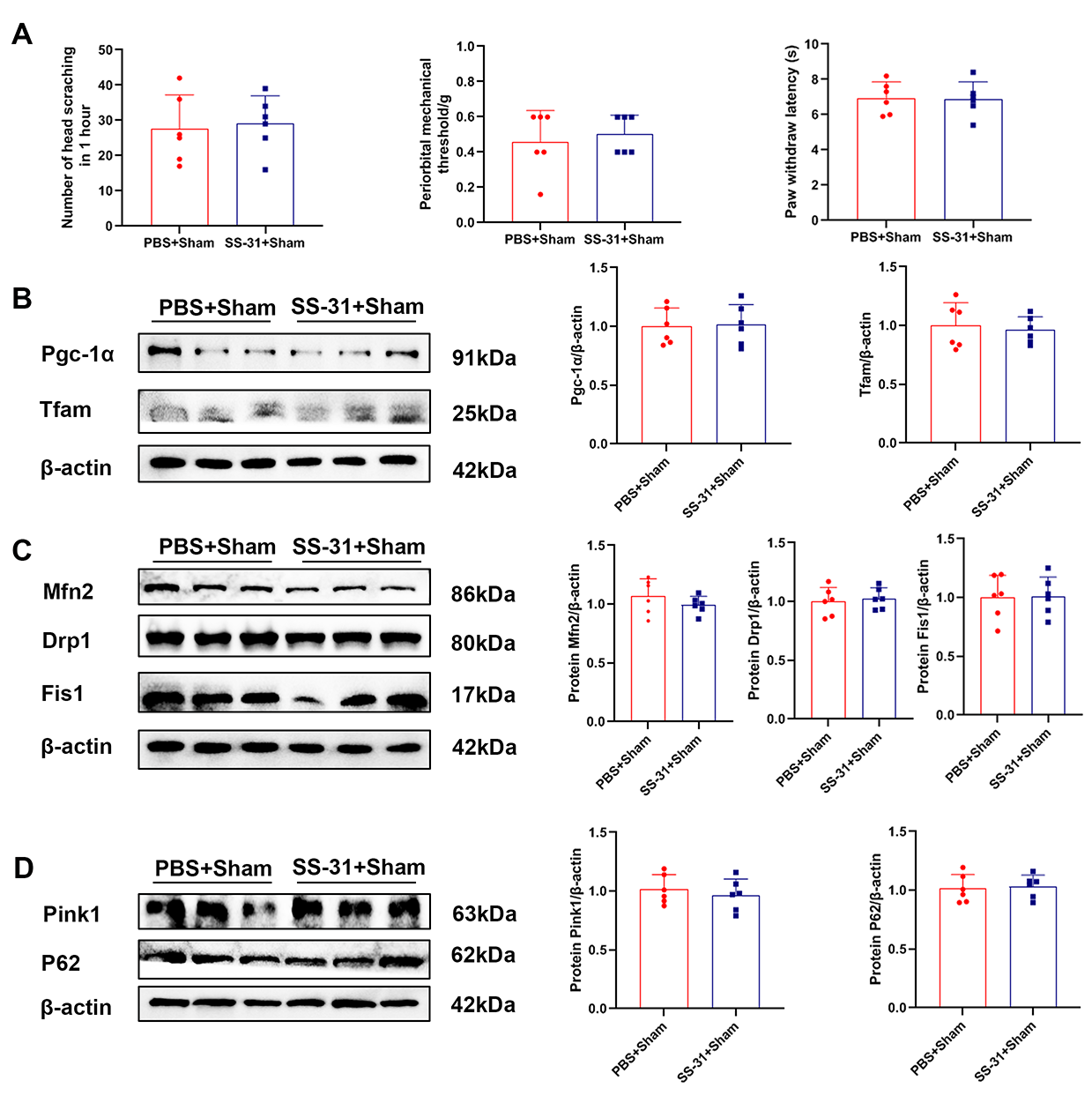
**

**Supplementary Fig. 4** SS-31 alone had no effect on behavioral and mitochondrial homeostasis of mice received sham treatment. Male C57BL/6 mice received PBS+sham or SS-31+sham treatment for 7 consecutive days, followed by behavioral tests and sacrifice to evaluate the effects of SS-31 on mitochondrial homeostasis. (A) The number of head scratching in 1-hour, periorbital mechanical threshold and paw withdrawal latency in different groups were recorded. n=6 per group; Student’s *t*-test. (B-D) Western blot analysis was used to asses expression levels of Pgc-1α, Tfam, Mfn2, Drp1, Fis1, P62 and Pink1 in different groups. n=6 per group; Student’s *t*-test. Data are represented as Mean ± SD.

**
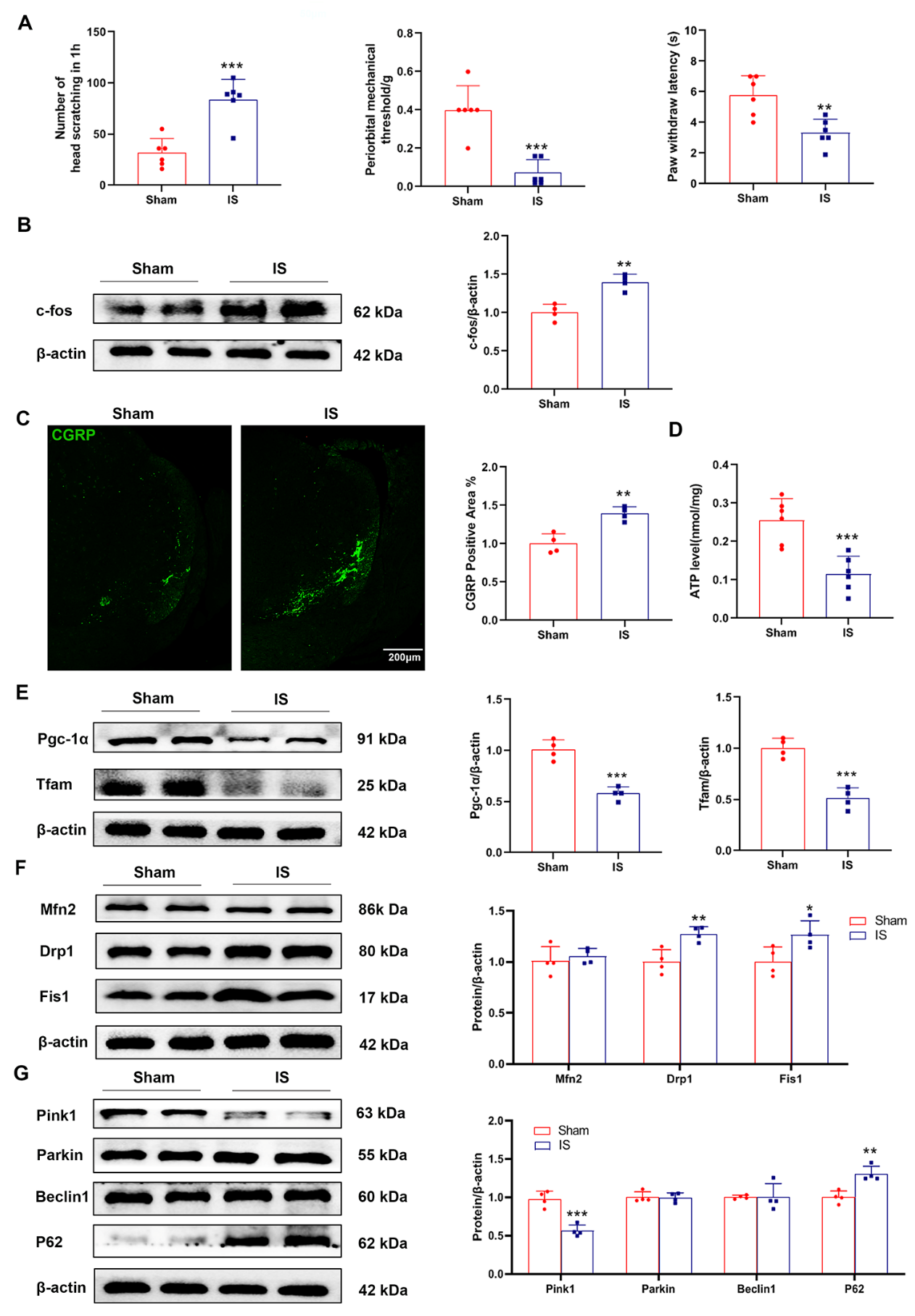
Supplementary Fig. 5** Nociceptive responses were activated, mitochondria were injured and mitochondrial homeostasis was unbalanced in an IS-induced headache female mouse model. Female C57BL/6 mice received sham or IS treatment for 7 consecutive days, followed by behavioral tests and sacrifice. (A) The number of head scratching in 1-hour (^***^*p*=0.0004), periorbital mechanical threshold (^***^*p*=0.0002) and paw withdrawal latency (^**^*p*=0.0036) in different groups were recorded. n=6 per group; Student’s *t*-test. (B) Representative immunoblots and quantification of the protein levels of c-fos in TNC (^***^*p*=0.0018). n=4 per group; Student’s *t*-test. (C) Immunofluorescence staining was used to examine the levels of CGRP in TNC (^***^*p*=0.0025). n=4 per group; Student’s *t*-test. (D) The levels of ATP (^***^*p*=0.0010) were detected and normalized by total protein concentrations in the TNC. n=6 per group; Student’s t-test. (E-G) Western blot analysis was used to asses expression levels of Pgc-1α (^***^*p*=0.0004), Tfam (^***^*p*=0.0004), Mfn2, Drp1 (^**^*p*=0.0075), Fis1 (^*^*p*=0.0392), P62 (^**^*p*=0.0034), Pink1 (^***^*p*=0.0007), Parkin and Beclin1 in different groups. n=4 per group; Student’s *t*-test. Data are represented as Mean ± SD; ^*^*p*<0.05, ^**^*p*<0.01 and ^***^*p*<0.001 as compared to sham group.
